# Red cell distribution width-to-albumin ratio and chronic kidney disease mortality in adults: A population-based NHANES 1999 to 2020 study

**DOI:** 10.1097/MD.0000000000044559

**Published:** 2026-06-12

**Authors:** Dong Wang, Zhengyang Zhu, Kejun Ren, Xiaowei Duan, Xulei Hu, Yong Lv, Hua Jin, Lei Zhang

**Affiliations:** a Department of Nephrology, The First Affiliated Hospital of Anhui University of Chinese Medicine, Hefei, Anhui Province, China.

**Keywords:** all-cause mortality, chronic kidney disease, neutrophil-to-lymphocyte ratio, NHANES, prognosis, red cell distribution width-to-albumin ratio

## Abstract

Chronic kidney disease (CKD) is associated with systemic inflammation and malnutrition, but prognostic tools integrating these pathways are lacking. This study investigated the red cell distribution width-to-albumin ratio (RAR) as a novel biomarker for predicting all-cause mortality in CKD patients. We conducted a retrospective cohort study using National Health and Nutrition Examination Survey 1999 to 2020 data (N = 40,133, including 6795 CKD cases). The RAR was derived from standardized laboratory measurements. Multivariable Cox regression with restricted cubic splines analyzed mortality risk, complemented by time-dependent receiver operating characteristic analysis and mediation analysis. Models were adjusted for 38 covariates spanning demographics, comorbidities, and biochemical parameters. Among 40,133 participants (6795 CKD cases) from the National Health and Nutrition Examination Survey (1999–2020), elevated RAR was independently linked to higher mortality risk (adjusted hazard ratio [HR] = 2.12, 95% confidence interval [CI] = 1.66–2.70, *P* < .001). Restricted cubic spline analysis revealed a nonlinear association, with an optimal RAR threshold of 4.26. Patients with RAR > 4.26 faced a 70% increased mortality risk compared to those ≤4.26 (HR = 1.70, 95% CI = 1.58–1.82, *P* < .001). RAR surpassed red cell distribution width alone in predicting 1-year mortality (area under the curve = 0.59, 95% CI = 0.57–0.60 vs 0.55, 95% CI = 0.54–0.57; *P* < .001). Subgroup analyses showed stronger mortality associations in males (HR = 2.39, 95% CI = 1.96–2.69), alcohol consumers (HR = 2.60, 95% CI = 2.20–3.01), and nonanemic individuals (HR = 2.45, 95% CI = 2.10–2.85; *P* interaction < .05). The neutrophil-to-lymphocyte ratio mediated 7.89% of RAR’s mortality risk. RAR, a composite biomarker reflecting erythrocyte instability and inflammation, provides robust prognostic value for CKD mortality. Its threshold (4.26) enables practical risk stratification, particularly in resource-limited settings. These findings support RAR’s integration into clinical workflows to improve personalized CKD management.

## 1. Introduction

The convergence of nephrology and immunometabolism has emerged as a pivotal research focus, given the growing recognition of chronic kidney disease (CKD) as a systemic disorder manifesting dysregulated inflammatory responses^[1]^ and increased cardiovascular mortality.^[2]^ Although renal replacement therapies have increased patient survival rates,^[3]^ they remain ineffective in addressing the persistent burden of premature mortality attributable to the multifactorial pathophysiology of CKD, particularly the triad comprising chronic inflammation,^[4]^ oxidative stress, and immune–hematopoietic axis dysfunction. Conventional biomarkers such as the estimated glomerular filtration rate (eGFR)^[5]^ and proteinuria quantification fail to adequately characterize the evolving interplay between erythrocyte homeostasis and immune activation that drives CKD progression. This critical knowledge gap highlights the imperative for multiparametric biomarkers^[6]^ that can simultaneously stratify mortality risk and elucidate underlying biological mechanisms. Red cell distribution width-to-albumin ratio (RAR), a multidimensional composite index quantifying erythrocyte heterogeneity through red cell distribution width (RDW) and reflecting inflammation–nutrition balance via serum albumin (ALB) levels, has demonstrated prognostic potential in cardiometabolic disorders.^[7]^ However, 3 fundamental questions remain unanswered regarding its application in CKD: whether RAR independently predicts mortality through neutrophil-to-lymphocyte ratio (NLR) and systemic immune-inflammatory index (SII) associated inflammatory cascades; how demographic variables mediate RAR interactions with immune–hematopoietic networks; and whether RAR offers incremental prognostic value beyond existing inflammatory indices. Addressing these knowledge gaps could transform CKD management in resource-constrained settings where current biomarker panels prove prohibitively expensive. Using data from the National Health and Nutrition Examination Survey (NHANES 1999–2020), this multicenter cohort study pioneers the systematic evaluation of RAR’s prognostic utility in 6795 clinically diagnosed CKD patients. Through restricted cubic spline (RCS) modeling,^[8]^ we established an optimal RAR threshold of 4.26 and demonstrated its superior predictive capacity over traditional indices (NLR/SII)^[9]^ via time-dependent receiver operating characteristic analysis.^[10]^ These findings necessitate a paradigm shift from compartmentalized risk assessment toward biomarkers integrating erythrocyte biology with immunometabolic regulation – a critical advancement enabling personalized therapeutic strategies in nephrology.

## 2. Materials and methods

### 2.1. Study population

**Figure 1.**
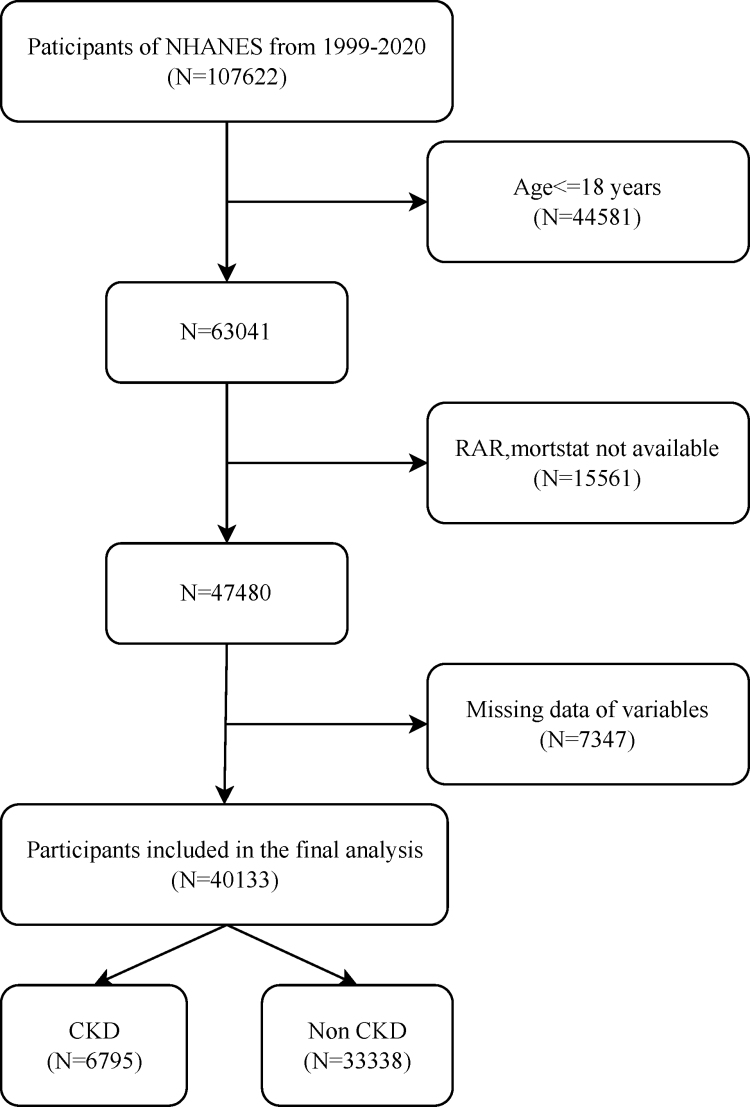
Flowchart of participant selection in NHANES (1999–2020). In the NHANES database, each participant was assigned a unique SEQN identifier. Across different data collection cycles, the same participant’s records were linked and consolidated using their respective SEQN identifiers. CKD = chronic kidney disease, NHANES = National Health and Nutrition Examination Survey, RAR = red cell distribution width-to-albumin ratio.


This investigation leveraged data from the NHANES,^[11]^ administered by the Centers for Disease Control and Prevention,^[12]^ employing standardized protocols, including structured interviews,^[13]^ physical examinations,^[14]^ 24-hour dietary recalls, and comprehensive laboratory assessments,^[15]^ to evaluate population health metrics.^[16]^ The study protocol received ethical approval from the National Center for Health Statistics Institutional Review Board under the Declaration of Helsinki guidelines, with written informed consent obtained from all participants. From the publicly accessible NHANES 1999 to 2020 datasets, we systematically identified 40,133 eligible adults (aged ≥18 years with complete survival records and essential covariates) through multistage screening of the initial 107,622 participants (Fig. 1). CKD diagnosis was established through 3 validated criteria^[17]^: physician-confirmed renal failure; persistent eGFR < 60 mL/min/1.73 m^2^ calculated via the Chronic Kidney Disease Epidemiology Collaboration (CKD-EPI) equation for ≥3 months; and urinary albumin–creatinine ratio (UACR) ≥30 mg/g. Biomarker quantification utilized Beckman Coulter hematology analyzers and standardized renal function assays, generating: RAR = RDW (%)/serum albumin (g/dL), eGFR = CKD-EPI^[18]^ creatinine equation (Inker et al; Equation 1), UACR = urinary albumin (mg/L)/urinary creatinine (g/L), NLR = neutrophils (10^3^/μL)/lymphocytes (10^3^/μL) × neutrophils (10^3^/μL)/lymphocytes (10^3^/μL),^[19]^ and vital status was ascertained through probabilistic linkage with the National Death Index, with follow-up duration calculated from baseline assessment to death, censoring, or study termination (December 31, 2019). The final analytical cohort (N = 40,133) included 6795 CKD cases classified per Kidney Disease: Improving Global Outcomes 2012 Clinical Practice Guidelines and 33,338 non-CKD controls. While the entire cohort provided prevalence estimates, survival analyses focused exclusively on the CKD subgroup via multivariable Cox regression.

$$\begin{array}{lll} eGFR= & \;141\times min{\left(\frac{Scr}{\kappa },1\right)}^{\alpha }\times max{\left(\frac{Scr}{\kappa },1\right)}^{-1.20}\; & \times 0.993^{Age}\times sex\;factor\times race\;factor \end{array}$$

Equation 1: Scr: Serum creatinine (mg/dL). κ: 0.7 (female), 0.9 (male). α: −0.329 (female), −0.411 (male). Sex factor: 1.018 (female), 1 (male). Race factor: 1.159 (black individuals), 1 (others).

### 2.2. Covariates

The demographic and clinical variables were categorized as follows: socioeconomic factors: age (continuous), sex (male/female), self-reported ethnicity (Mexican American, non-Hispanic White, non-Hispanic Black, other), marital status (married vs other [widowed/divorced/separated/never married/cohabiting]), income–poverty ratio (PIR: ≤1.0 vs >1.0), and education level (less than high school/high school or equivalent/college or above); lifestyle factors: smoking status (current/former/never), alcohol consumption (yes/no), physical activity level (low physical activity/high physical activity); and comorbidity profile: physician-diagnosed preexisting conditions, including anemia, hypertension, diabetes mellitus, and hyperlipidemia. The biochemical parameters quantified through standardized assays included the following serum concentrations: sodium (Na), potassium (K), chloride (Cl), bicarbonate (HCO_3_^−^), total calcium (Ca), creatinine (Scr), ALB, total protein (TP), glucose (Glu), triglycerides (TG), high-density lipoprotein (HDL), uric acid (UA), alanine transaminase (ALT), aspartate transaminase (AST), and gamma-glutamyl transferase. Hematological parameters included the basophil percentage (BaP), lymphocyte percentage (LymP), monocyte percentage (MonP), eosinophil percentage (EoP), absolute lymphocyte count (Lym), monocyte count (Mon), eosinophil count (Eo), segmented neutrophil count (Segne), basophil count (Ba), red blood cell count (RBC), hemoglobin (Hg), hematocrit (Hem), mean corpuscular hemoglobin (MCH), mean corpuscular hemoglobin concentration (MCHC), mean platelet volume (MPV), and mean corpuscular volume. All variables were operationally defined according to NHANES documentation protocols.

### 2.3. Statistical analysis

We accounted for the complex survey design of NHANES by incorporating sample weights, clustering, and stratification. To achieve nationally representative estimates, the original survey weights were adjusted and utilized in the analysis, taking into account the appropriate adjustments, Statistical analyses were performed using R software (version 4.3.0; R Foundation for Statistical Computing). The nhanesR package (version 0.9.4.8; The Comprehensive R Archive Network) was used to glean clinical data from the NHANES database (cycles from 1999 to 2020; https://wwwn.cdc.gov/nchs/nhanes/Default.aspx). Student *t* tests were adopted when continuous variables followed a Gaussian distribution on analysis of variance. Otherwise, the Mann–Whitney *U* test was applied. The chi-squared test was used to evaluate variable factors.

To better explore the relationship between CKD prevalence and RAR stratification, we divided them into 4 groups (Q1, Q2, Q3, and Q4) according to the quartile data of RAR. The ranges of RAR (g/dL) in Q1 to Q4 are (2.02, 2.82), (2.82, 3.05), (3.05, 3.38), and (3.38, 12.08), respectively. Logistic regression was used to determine CKD and healthy group odds ratio (OR), while Cox regression was used to determine hazard ratio (HR). Both OR and HR were calculated with corresponding 95% confidence intervals (CIs).^[20]^ To better explore the relationship between RAR and CKD, 4 models were employed in the adjustment. Model 1: adjusted with none; Model 2: adjusted with sex, age; Model 3: adjusted with sex, ethnicity, marital status, PIR, smoking, education level, drinking, physical activity, anemia, hypertension, diabetes mellitus, hyperlipidemia, age; Model 4: adjusted with sex, ethnicity, marital status, PIR, smoking, education level, drinking, physical activity, anemia, hypertension, diabetes mellitus, hyperlipidemia, ALT, AST, Ca, HCO_3_, gamma-glutamyl transferase, Glu, TP, TG, UA, Na, K, Cl, MonP, EoP, BaP, Mon, Eo, Ba, RBC, Hem, MCH, MCHC, MPV, HDL, body mass index, age.

RCS models with 4 knots were employed to examine potential nonlinear associations between RAR and mortality risk in patients with CKD, while simultaneously determining the optimal RAR threshold predictive of all-cause mortality. Kaplan–Meier curves were generated to estimate survival probabilities, with between-group differences assessed through log-rank tests. Subgroup analyses stratified by demographic factors (age, sex, ethnicity),^[21]^ socioeconomic status (educational level, marital status, PIR), lifestyle factors (drinking, smoking, physical activity), and clinical comorbidities (diabetes, hyperlipidemia, anemia, hypertension) were conducted to evaluate RAR-mortality associations and test for potential interaction effects. Statistical significance was defined as a two-tailed *P* value <.05.

Causal mediation analysis is a key technique for disentangling direct and indirect causal effects.^[22]^ In this population-based study, we implemented this framework to systematically assess inflammatory biomarkers – specifically NLR and SII – as potential mediators between RAR and CKD progression. Leveraging NHANES survey data, we accounted for complex sampling designs through the svydesign function in R’s survey package. Our 3-stage analytical protocol comprised: establishing baseline RAR-CKD associations; evaluating RAR-mediator and mediator-CKD relationships; quantifying mediation effects using Cox proportional hazards models adjusted for RAR. Quasi-Bayesian inference with 1000 Monte Carlo simulations generated robust CIs.

## 3. Results

### 3.1. Characteristics of the study population

As shown in Table 1, this retrospective cohort study included 40,133 participants (6795 CKD patients; 33,338 non-CKD controls), representing 224,440,284 weighted Americans. The cohort included 20,686 females (51.54%) and 19,447 males (48.46%), with a mean age of 47.07 years. A total of 11,467 participants (28.57%) were aged >60 years. Compared with the non-CKD group, the CKD group presented a greater retinol-binding protein-to-albumin ratio (3.19 ± 0.01 vs 2.98 ± 0.00, *P* < .001). Six of the 50 analyzed variables (HCO_3_, TP, Na, MCHC, MPV, and HDL) were not significantly different. CKD patients presented higher prevalence rates of anemia, diabetes mellitus, hypertension, and dyslipidemia than non-CKD controls did (*P* < .001). The CKD population had greater proportions of older individuals, females, those with lower educational attainment, and those with lower socioeconomic status. CKD patients reported higher rates of smoking, physical activity, and drinking (*P* < .001). The RAR quartiles (RARQs) are described, and the general RAR quartile (RARQ) data are summarized in Table S1, Supplemental Digital Content. The incidence of CKD among RARQ patients was 7.04%, 9.17%, 14.68%, and 22.08%, respectively; the DM incidence increased as RAR increased. With increasing RAR levels, multiple variables, including metabolic parameters (body mass index, UA), inflammatory markers (SII, NLR), renal injury indicators (Ualb, UACR, Scr), and the prevalence of comorbid conditions, including hypertension, diabetes, and anemia, significantly increased. Populations characterized by advanced age, female gender, non-Hispanic Black ethnicity, and lower socioeconomic status (poor and less than high school) are substantially more represented in the highest RARQ group.

**Table 1:** Basic demographic data of CKD and non-CKD subjects.

Variable	Total (n = 40,133)	Non-CKD (n = 33,338)	CKD (n = 6795)	Statistic	*P*
ALB, mean (SE)	4.33 (0.01)	4.35 (0.01)	4.22 (0.01)	*t* = −20.35	<.001
ALT, mean (SE)	25.77 (0.16)	25.95 (0.17)	24.59 (0.33)	*t* = −3.80	<.001
AST, mean (SE)	25.47 (0.11)	25.32 (0.12)	26.46 (0.30)	*t* = 3.59	<.001
Ca, mean (SE)	9.44 (0.01)	9.44 (0.01)	9.47 (0.01)	*t* = 3.26	.001
HCO_3_, mean (SE)	24.71 (0.06)	24.71 (0.07)	24.75 (0.07)	*t* = 0.89	.377
GGT, mean (SE)	27.88 (0.26)	27.03 (0.25)	33.47 (1.04)	*t* = 6.10	<.001
Glu, mean (SE)	97.45 (0.23)	95.03 (0.21)	113.25 (0.75)	*t* = 24.37	<.001
TP, mean (SE)	71.96 (0.10)	71.98 (0.09)	71.84 (0.14)	*t* = −1.44	.154
TG, mean (SE)	146.84 (1.13)	143.52 (1.19)	168.52 (2.36)	*t* = 10.08	<.001
UA, mean (SE)	5.38 (0.01)	5.29 (0.01)	5.92 (0.03)	*t* = 21.15	<.001
Scr, mean (SE)	0.85 (0.00)	0.82 (0.00)	1.08 (0.01)	*t* = 24.26	<.001
Na, mean (SE)	139.19 (0.06)	139.19 (0.06)	139.18 (0.07)	*t* = −0.14	.886
K, mean (SE)	4.01 (0.01)	4.00 (0.01)	4.08 (0.01)	*t* = 11.16	<.001
Cl, mean (SE)	103.50 (0.08)	103.56 (0.09)	103.10 (0.10)	*t* = −7.79	<.001
LymP, mean (SE)	29.97 (0.08)	30.29 (0.08)	27.85 (0.16)	*t* = −14.70	<.001
MonP, mean (SE)	7.97 (0.02)	7.95 (0.03)	8.06 (0.05)	*t* = 2.15	.033
EoP, mean (SE)	2.82 (0.01)	2.80 (0.01)	2.91 (0.04)	*t* = 2.82	.006
BaP, mean (SE)	0.70 (0.01)	0.70 (0.01)	0.72 (0.01)	*t* = 2.33	.021
Lym, mean (SE)	2.13 (0.01)	2.14 (0.01)	2.07 (0.02)	*t* = −3.70	<.001
Mon, mean (SE)	0.56 (0.00)	0.56 (0.00)	0.59 (0.00)	*t* = 7.46	<.001
Segne, mean (SE)	4.34 (0.02)	4.29 (0.02)	4.62 (0.04)	*t* = 10.21	<.001
Eo, mean (SE)	0.20 (0.00)	0.20 (0.00)	0.21 (0.00)	*t* = 5.10	<.001
Ba, mean (SE)	0.04 (0.00)	0.04 (0.00)	0.05 (0.00)	*t* = 4.52	<.001
RBC, mean (SE)	4.70 (0.01)	4.72 (0.01)	4.57 (0.01)	*t* = −16.68	<.001
Hg, mean (SE)	14.32 (0.02)	14.38 (0.02)	13.94 (0.03)	*t* = −18.17	<.001
Hem, mean (SE)	42.15 (0.07)	42.30 (0.07)	41.17 (0.09)	*t* = −16.13	<.001
MCH, mean (SE)	89.83 (0.07)	89.76 (0.07)	90.29 (0.13)	*t* = 4.86	<.001
MCHC, mean (SE)	30.53 (0.03)	30.52 (0.03)	30.58 (0.06)	*t* = 1.38	.170
RDW, mean (SE)	12.92 (0.01)	12.85 (0.01)	13.36 (0.02)	*t* = 20.43	<.001
MPV, mean (SE)	8.18 (0.01)	8.17 (0.01)	8.21 (0.02)	*t* = 1.90	.059
HDL, mean (SE)	53.00 (0.22)	53.04 (0.22)	52.70 (0.36)	*t* = −1.04	.299
Ualb, mean (SE)	32.03 (1.47)	9.29 (0.08)	180.64 (10.71)	*t* = 16.02	<.001
BMI, mean (SE)	28.48 (0.07)	28.32 (0.07)	29.55 (0.14)	*t* = 9.31	<.001
Age, mean (SE)	45.55 (0.20)	43.48 (0.19)	59.12 (0.39)	*t* = 44.62	<.001
UACR, mean (SE)	30.52 (1.56)	7.56 (0.06)	180.56 (11.30)	*t* = 15.33	<.001
EGFR, mean (SE)	103.91 (0.27)	107.58 (0.25)	79.94 (0.60)	*t* = −52.53	<.001
RAR, mean (SE)	3.01 (0.00)	2.98 (0.00)	3.19 (0.01)	*t* = 25.42	<.001
NLR, mean (SE)	2.22 (0.01)	2.17 (0.01)	2.55 (0.03)	*t* = 14.29	<.001
SII, mean (SE)	565.39 (3.36)	554.95 (3.24)	636.72 (9.39)	*t* = 8.83	<.001
Sex, n (%)				χ^2^ = 92.77	<.001
Male	19,447 (48.37)	16,284 (49.31)	3163 (42.23)		
Female	20,686 (51.63)	17,054 (50.69)	3632 (57.77)		
Ethnicity, n (%)				χ^2^ = 20.55	<.001
Mexican American	7531 (8.15)	6438 (8.33)	1093 (7.04)		
Non-Hispanic White	17,452 (68.88)	14,170(68.80)	3282 (69.40)		
Non-Hispanic Black	8253 (10.72)	6787 (10.53)	1466 (11.96)		
Other	6897 (12.25)	5943 (12.35)	954 (11.60)		
Marital status, n (%)				χ^2^ = 54.44	<.001
Married	22,600 (60.57)	19,014 (61.27)	3586 (55.97)		
Other (widowed, divorced, separated, never married, living with a partner)	17,533 (39.43)	14,324 (38.73)	3209 (44.03)		
PIR, n (%)				χ^2^ = 34.91	<.001
Poor	8159 (13.67)	6745 (13.27)	1414 (16.26)		
Not poor	31,974 (86.33)	26,593(86.73)	5381 (83.74)		
Smoking, n (%)				χ^2^ = 34.73	<.001
No	23,056 (54.90)	19,554(55.47)	3502 (51.16)		
Yes	17,077 (45.10)	13,784(44.53)	3293 (48.84)		
Education level, n (%)				χ^2^ = 236.60	<.001
Less than high school	11,336 (18.84)	8943 (17.79)	2393 (25.67)		
High school or equivalent	9486 (23.87)	7851 (23.62)	1635 (25.52)		
College or above	19,311 (57.29)	16,544 (58.59)	2767 (48.80)		
Drinking, n (%)				χ^2^ = 150.31	<.001
No	15,375 (31.23)	12,500 (30.12)	2875 (38.48)		
Yes	24,758 (68.77)	20,838 (69.88)	3920 (61.52)		
Physical activity, n (%)				χ^2^ = 417.46	<.001
Low physical activity	18,538 (43.67)	14,460 (41.69)	4078 (56.60)		
High physical activity	21,595 (56.33)	18,878 (58.31)	2717 (43.40)		
Anemia, n (%)				χ^2^ = 538.05	<.001
No	36,377 (93.73)	30,820 (94.83)	5557 (86.56)		
Yes	3756 (6.27)	2518 (5.17)	1238 (13.44)		
Hypertension, n (%)				χ^2^ = 2301.71	<.001
No	24,776 (65.81)	22,650 (70.25)	2126 (36.77)		
Yes	15,357 (34.19)	10,688 (29.75)	4669 (63.23)		
Diabetes mellitus, n (%)				χ^2^ = 2214.66	<.001
No	33,723 (88.26)	29,471 (91.22)	4252 (68.94)		
Yes	6410 (11.74)	3867 (8.78)	2543 (31.06)		
Hyperlipidemia, n (%)				χ^2^ = 362.01	<.001
No	11,960 (29.03)	10,683 (30.72)	1277 (18.01)		
Yes	28,173 (70.97)	22,655 (69.28)	5518 (81.99)		

All estimates accounted for complex survey designs.

ALB = albumin, ALT = alanine transaminase, AST = aspartate transaminase, Ba = basophil count, BAP = basophil percentage, BMI = body mass index, Ca = total calcium, CKD = chronic kidney disease, Cl = chloride, eGFR = estimated glomerular filtration rate, Eo = eosinophil count, EoP = eosinophil percentage, GGT = gamma-glutamyl transferase, Glu = glucose, HCO_3_^−^ = bicarbonate, HDL = high-density lipoprotein, Hem = haematocrit, Hg = haemoglobin, K = potassium, Lym = absolute lymphocyte count, LymP = lymphocyte percentage, MCH = mean corpuscular haemoglobin, MCHC = mean corpuscular hemoglobin concentration, MCV = mean corpuscular volume, Mon = monocyte count, MonP = monocyte percentage, MPV = mean platelet volume, Na = sodium, NLR = neutrophil-to-lymphocyte ratio, PIR = poverty-to-income ratio, RAR = red cell distribution width-to-albumin ratio, RBC = red blood cell count, RDW = red cell distribution width, Scr = creatinine, Senge = segmented neutrophil count, SII = systemic immune-inflammatory index, *t* = *t* test, TG = triglycerides, TP = total protein, UA = uric acid, UACR = urinary albumin–creatinine ratio, χ^2^ = chi-square test.

### 3.2. The correlation between RAR and CKD incidence

This multivariable analysis demonstrated that RAR was significantly associated with CKD risk (Table 2), with an unadjusted OR of 2.57 (95% CI = 2.39–2.77, *P* < .001) in Model 1, which persisted after full adjustment in Model 4 (OR = 1.80, 95% CI = 1.62–2.00, *P* < .001). RARQ analysis revealed a dose-dependent risk gradient: Q2 had an OR of 1.33 (95% CI = 1.13–1.57, *P* < .001) in Model 1 but became nonsignificant after adjustment (Model 3: OR = 0.93, 95% CI = 0.78–1.09, *P* = .365), whereas Q3/Q4 remained significant in Model 4 (OR = 1.43, 95% CI = 1.21–1.68; OR = 1.88, 95% CI = 1.59–2.23, *P* < .001). Among inflammatory markers, RDW decreased from OR = 1.33 (95% CI = 1.29–1.37, *P* < .001) in Model 1 to 1.15 (95% CI = 1.11–1.19, *P* < .001) in Model 4; NLR attenuated from 1.27 (95% CI = 1.23–1.31, *P* < .001) to 1.16 (95% CI = 1.11–1.21, *P* < .001). SII had a minimal effect (Model 4: OR = 1.01, 95% CI = 1.01–1.01) but maintained statistical significance (*P* < .001). These findings identify RAR, RDW, and the NLR as independent CKD risk predictors and the stability of RAR’s effect size following extensive adjustment for confounders underscores its superior independent predictive capacity for CKD compared with other variables, with RARQ stratification (88% elevated risk in Q4 vs Q1), suggesting clinical utility.

**Table 2:** Logistic regression.

Variables	CKD							
	OR (95% CI)*	*P*	OR (95% CI)†	*P*	OR (95% CI)‡	*P*	OR (95% CI)§	*P*
RAR	2.57 (2.39–2.77)	<.001	1.95 (1.81–2.10)	<.001	1.47 (1.34–1.61)	<.001	1.80 (1.62–2.00)	<.001
RARQ								
Q1	1.00 (Reference)		1.00 (Reference)		1.00 (Reference)		1.00 (Reference)	
Q2	1.33 (1.13–1.57)	<.001	0.95 (0.80–1.12)	.527	0.93 (0.78–1.09)	.365	1.04 (0.88–1.22)	.668
Q3	2.27 (1.95–2.66)	<.001	1.30 (1.12–1.52)	<.001	1.19 (1.02–1.39)	.030	1.43 (1.21–1.68)	<.001
Q4	3.74 (3.25–4.31)	<.001	1.95 (1.70–2.24)	<.001	1.46 (1.26–1.70)	<.001	1.88 (1.59–2.23)	<.001
RDW	1.33 (1.29–1.37)	<.001	1.22 (1.19–1.25)	<.001	1.12 (1.08–1.15)	<.001	1.15 (1.11–1.19)	<.001
NLR	1.27 (1.23–1.31)	<.001	1.19 (1.15–1.23)	<.001	1.18 (1.14–1.21)	<.001	1.16 (1.11–1.21)	<.001
SII	1.01 (1.01–1.01)	<.001	1.01 (1.01–1.01)	<.001	1.01 (1.01–1.01)	<.001	1.01 (1.01–1.01)	<.001

ALT = alanine transaminase, AST = aspartate transaminase, Ba = basophil count, BAP = basophil percentage, BMI = body mass index, Ca = total calcium, CI = confidence interval, CKD = chronic kidney disease, Cl = chloride, Eo = eosinophil count, EoP = eosinophil percentage, GGT = gamma-glutamyl transferase, Glu = glucose, HCO_3_^−^ = bicarbonate, HDL = high-density lipoprotein, Hem = haematocrit, Hg = haemoglobin, K = potassium, Lym = absolute lymphocyte count, LymP = lymphocyte percentage, MCH = mean corpuscular haemoglobin, MCHC = mean corpuscular hemoglobin concentration, MCV = mean corpuscular volume, Mon = monocyte count, MonP = monocyte percentage, MPV = mean platelet volume, Na = sodium, NLR = neutrophil-to-lymphocyte ratio, OR = odds ratio, PIR = poverty-to-income ratio, RAR = red cell distribution width-to-albumin ratio, RBC = red blood cell count, RDW = red cell distribution width, Scr = creatinine, Senge = segmented neutrophil count, SII = systemic immune-inflammatory index, TG = triglycerides, TP = total protein, UA = uric acid.

* Model 1: Crude.

† Model 2: Sex and age.

‡ Model 3: Adjust: Sex, ethnicity, marital status, PIR, smoking, education level, drinking, physical activity, anemia, hypertension, diabetes mellitus, hyperlipidemia, and age.

§ Model 4: Adjust: Sex, ethnicity, marital status, PIR, smoking, education level, drinking, physical activity, anemia, hypertension, diabetes mellitus, hyperlipidemia, ALT, AST, Ca, HCO_3_, GGT, Glu, TP, TG, UA, Na, K, Cl, MonP, EoP, BaP, Mon, Eo, Ba, RBC, Hem, MCH, MCHC, MPV, HDL, BMI, and age.

### 3.3. Sensitivity analysis stratified by sex

The sex-stratified sensitivity analysis revealed distinct patterns in CKD risk associations. Comprehensive details are summarized in Table S2, Supplemental Digital Content. In the crude model, males presented a stronger baseline association (HR = 2.29, 95% CI = 1.96–2.69, *P* < .001) than females did (HR = 1.66, 95% CI = 1.51–1.84, *P* < .001), with the highest risk observed in the fourth quartile (Q4) for both sexes (males: HR = 4.84; females: HR = 2.98). Age adjustment attenuated the risk in males (HR = 1.90, 95% CI = 1.62–2.23) but paradoxically increased it in females (HR = 1.92, 95% CI = 1.69–2.18). Further adjustment for sociodemographic, behavioral, and clinical covariates reduced the male risk estimates (Model 3: HR = 1.76; Model 4: HR = 1.98), whereas the female risk estimates remained stable (Model 3: HR = 1.77; Model 4: HR = 1.90). Across all the models, Q4 consistently presented the highest risk in both sexes (males: HR = 2.30–4.84; females: HR = 2.42–2.98, all *P* < .001).

### 3.4. Association of RAR with mortality outcomes

**Figure 2.**
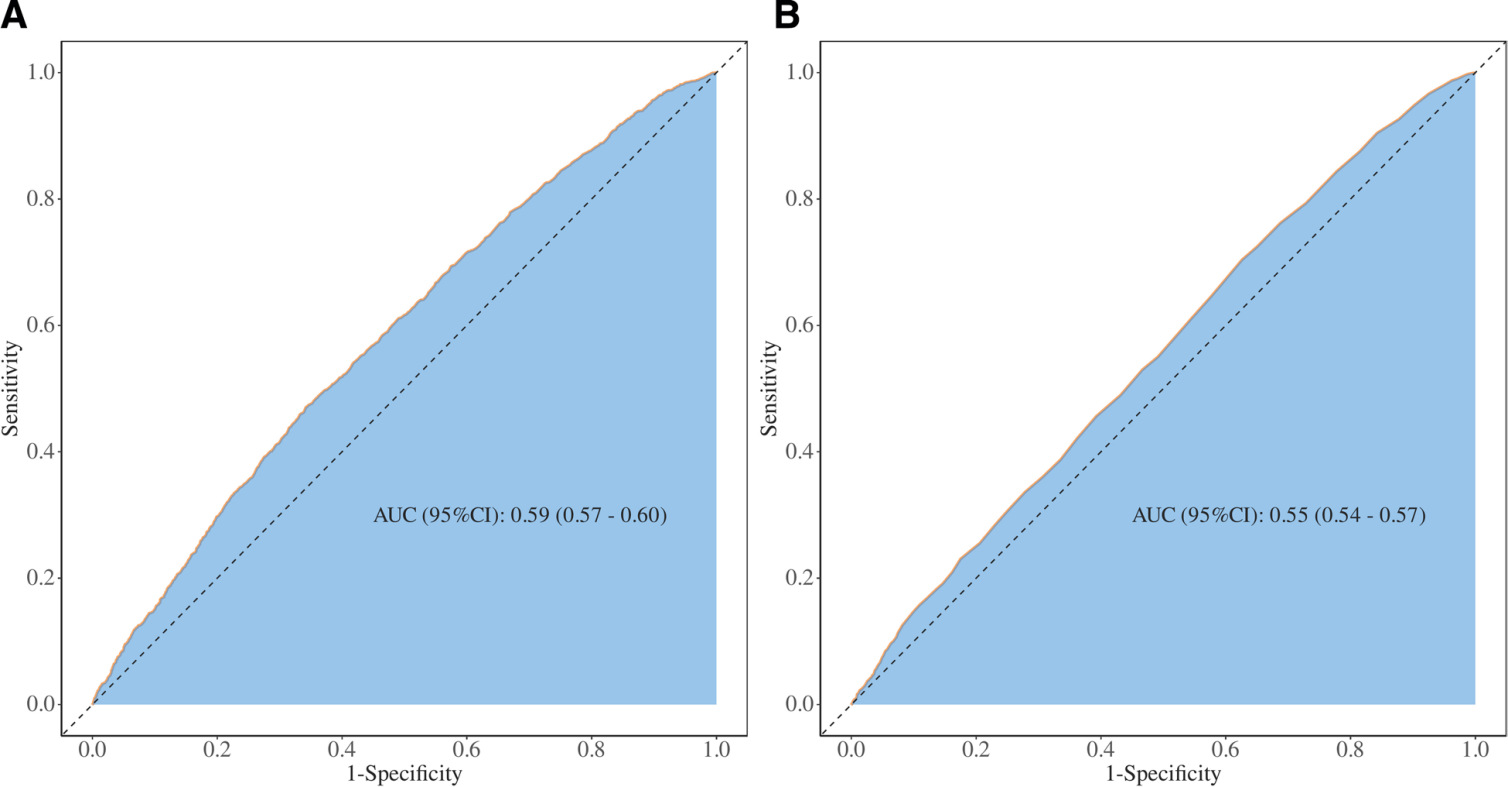
ROC analysis of RAR and RDW in clinical prognosis. Panel (A) presents the ROC analysis of RAR. Panel (B) presents the ROC analysis of RDW. AUC = area under the curve, CI = confidence interval, RAR = red cell distribution width-to-albumin ratio, RDW = red cell distribution width, ROC = receiver operating characteristic.

During a median follow-up period of 93 months, 2563 deaths (37.72%) were observed among the 6795 participants with CKD. The characteristics of these participants are presented in Table S3, Supplemental Digital Content. In a cohort of 6795 CKD patients, deceased individuals presented significantly poorer renal function (lower eGFR, higher Scr), elevated systemic inflammation (NLR, SII), metabolic dysregulation (hyperglycemia, hyperuricemia), greater comorbidity burden (hypertension, diabetes, anemia), and adverse lifestyle profiles (smoking, physical activity) than survivors did, with statistically significant differences (*P* < .05). In the unadjusted model (Model 1), RAR exhibited superior predictive value for CKD prognosis compared with RDW (Table 3). Each 1-unit increase in RAR was associated with an 82% greater risk of all-cause mortality in the crude model (HR = 1.82, 95% CI = 1.67–1.99, *P* < .01), whereas RDW was associated with only an 18% increase in risk (HR = 1.18, 95% CI = 1.15–1.22, *P* < .01). This disparity persisted in the fully adjusted model (Model 4: RAR HR = 1.87, 95% CI = 1.64–2.13; RDW HR = 1.17, 95% CI = 1.12–1.22, *P* < .01). Nonoverlapping CIs across all the models (e.g., Model 1: RAR 1.67–1.99 vs RDW 1.15–1.22) and stable hazard ratios for RAR (range: 1.76–1.90) underscore its robustness as an independent predictor. Receiver operating characteristic analysis demonstrated that the RAR significantly outperformed the RDW alone in disease risk stratification. The area under the curve (AUC) of the RAR was 0.59 (95% CI = 0.57–0.60), which was significantly greater than that of the RDW (0.55 [95% CI = 0.54–0.57]; Fig. 2). The significance of the AUC difference (ΔAUC = 0.04, *P* = .021) was confirmed by DeLong test, highlighting the superior predictive value of RAR as a composite biomarker integrating inflammatory and nutritional status.

**Table 3:** Single- and multiple-variable regulation of the Cox regression model.

Variables	All-cause mortality							
	HR (95% CI)*	*P*	HR (95% CI)†	*P*	HR (95% CI)‡	*P*	HR (95% CI)§	*P*
RAR	1.82 (1.67–1.99)	<.001	1.90 (1.72–2.10)	<.001	1.76 (1.59–1.95)	<.001	1.87 (1.64–2.13)	<.001
RARQ								
Q1	1.00 (Reference)		1.00 (Reference)		1.00 (Reference)		1.00 (Reference)	
Q2	1.78 (1.53–2.06)	<.001	1.30 (1.12–1.52)	<.001	1.29 (1.09–1.52)	.002	1.39 (1.18–1.64)	<.001
Q3	2.32 (1.99–2.70)	<.001	1.48 (1.25–1.74)	<.001	1.38 (1.16–1.65)	<.001	1.57 (1.31–1.88)	<.001
Q4	3.76 (3.26–4.32)	<.001	2.57 (2.20–3.01)	<.001	2.24 (1.90–2.65)	<.001	2.57 (2.16–3.06)	<.001
RDW	1.18 (1.15–1.22)	<.001	1.20 (1.16–1.24)	<.001	1.17 (1.12–1.21)	<.001	1.17 (1.12–1.22)	<.001
NLR	1.23 (1.20–1.26)	<.001	1.18 (1.14–1.21)	<.001	1.17 (1.14–1.20)	<.001	1.16 (1.13–1.19)	<.001
SII	1.01 (1.01–1.01)	<.001	1.01 (1.01–1.01)	<.001	1.01 (1.01–1.01)	<.001	1.01 (1.01–1.01)	<.001

ALT = alanine transaminase, AST = aspartate transaminase, Ba = basophil count, BAP = basophil percentage, BMI = body mass index, Ca = total calcium, CI = confidence interval, CKD = chronic kidney disease, Cl = chloride, Eo = eosinophil count, EoP = eosinophil percentage, GGT = gamma-glutamyl transferase, Glu = glucose, HCO_3_^−^ = bicarbonate, HDL = high-density lipoprotein, Hem = haematocrit, Hg = haemoglobin, HR = hazard ratio, K = potassium, Lym = absolute lymphocyte count, LymP = lymphocyte percentage, MCH = mean corpuscular haemoglobin, MCHC = mean corpuscular hemoglobin concentration, MCV = mean corpuscular volume, Mon = monocyte count, MonP = monocyte percentage, MPV = mean platelet volume, Na = sodium, NLR = neutrophil-to-lymphocyte ratio, PIR = poverty-to-income ratio, RAR = red cell distribution width-to-albumin ratio, RBC = red blood cell count, RDW = red cell distribution width, Scr = creatinine, Senge = segmented neutrophil count, SII = systemic immune-inflammatory index, TG = triglycerides, TP = total protein, UA = uric acid.

* Model 1: Crude.

† Model 2: Adjust: Sex, age.

‡ Model 3: Sex, ethnicity, marital status, PIR, smoking, education level, drinking, physical activity, anemia, hypertension, diabetes mellitus, hyperlipidemia, and age.

§ Model 4: Sex, ethnicity, marital status, PIR, smoking, education level, drinking, physical activity, anemia, hypertension, diabetes mellitus, hyperlipidemia, ALT, AST, Ca, HCO_3_, GGT, Glu, TP, TG, UA, Na, K, Cl, MonP, EoP, BaP, Mon, Eo, Ba, RBC, Hem, MCHC, MCHC, MPV, HDL, BMI, and age.

### 3.5. Nonlinear associations and survival rates

**Figure 3.**
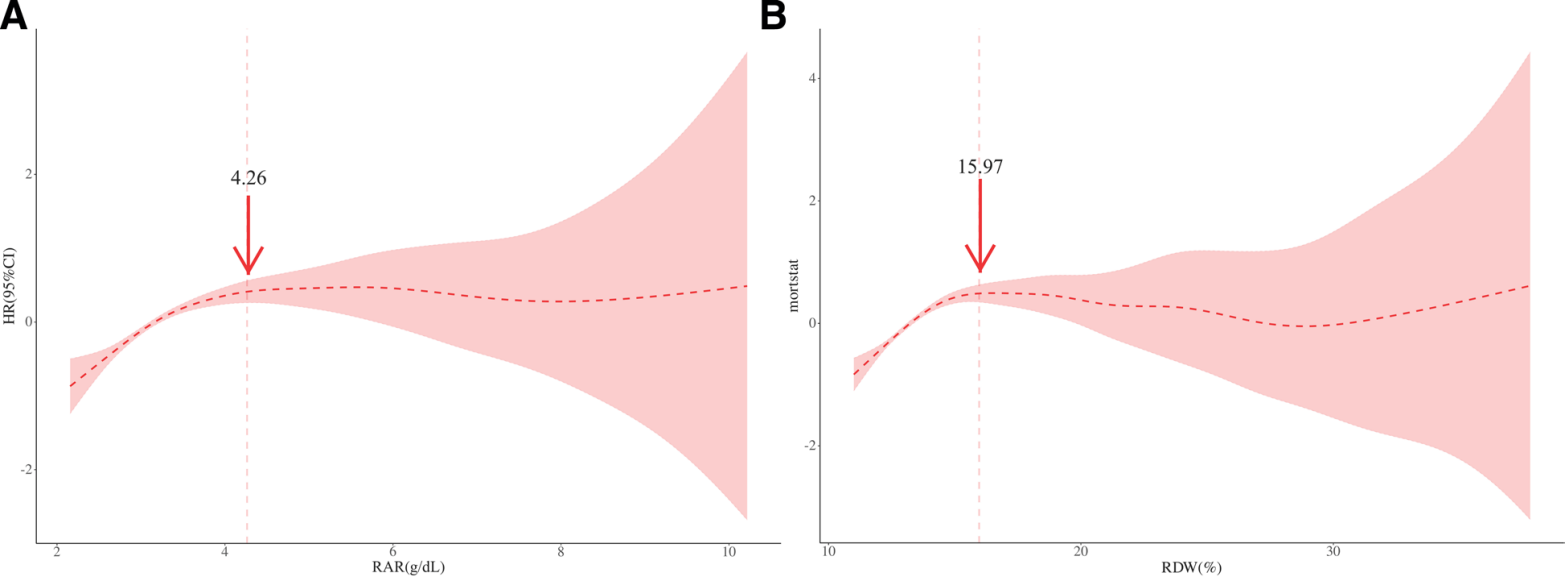
RCS analysis of RAR and RDW for all-cause mortality. (A) RCS analysis for RAR. (B) RCS analysis for RDW. CI = confidence interval, HR = hazard ratio, RAR = red cell distribution width-to-albumin ratio, RCS = restricted cubic spline, RDW = red cell distribution width.

**Figure 4.**
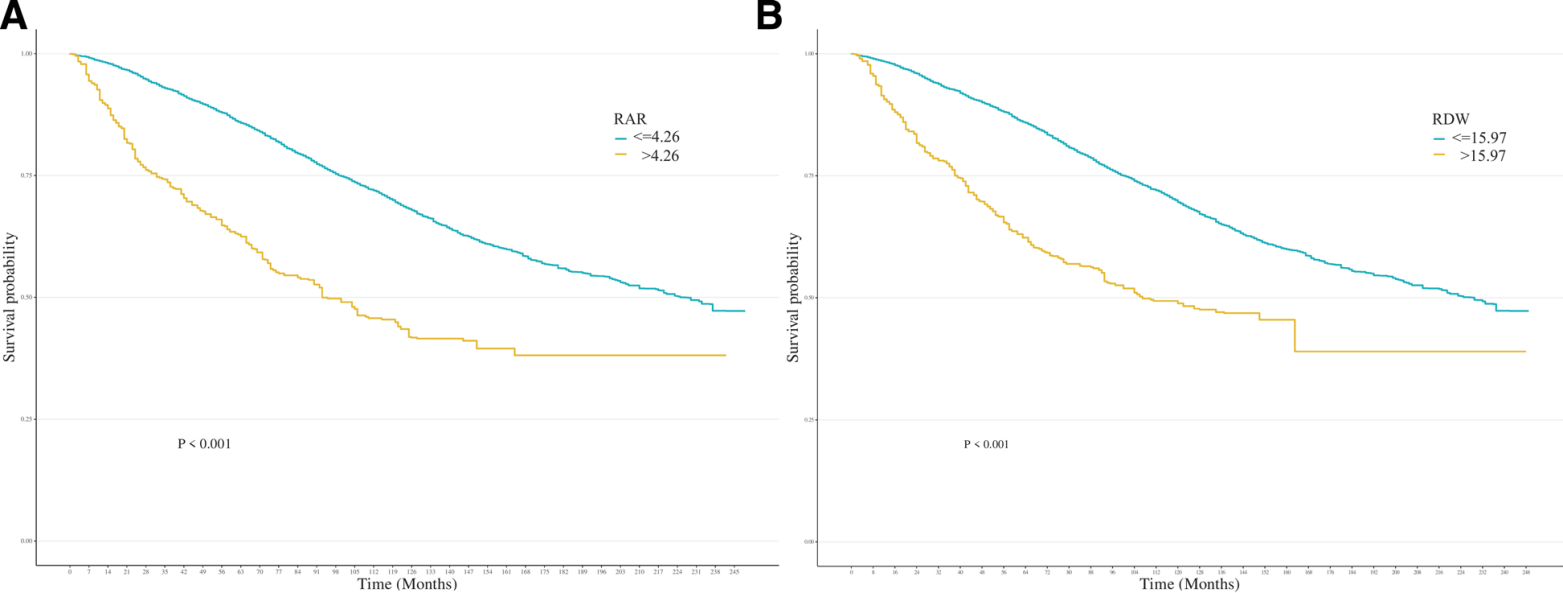
Kaplan–Meier curve construction. (A) Kaplan–Meier curves for RAR. (B) Kaplan–Meier curves for RDW. RAR = red cell distribution width-to-albumin ratio, RDW = red cell distribution width.

RCS analysis revealed nonlinear associations between RAR/RDW and all-cause mortality, with optimal cutoff values of 4.26 for RAR and 15.97 for RDW (Fig. 3). In CKD patients, both biomarkers demonstrated prognostic significance for adverse outcomes, showing progressively elevated HR with increasing values. Stratification on the basis of these thresholds classified CKD patients into high-RAR (>4.26) and high-RDW (>15.97) subgroups. The overall HR for RAR significantly surpassed that of RDW (HR = 1.70, 95% CI = 1.58–1.82, *P* < .001 vs HR = 1.13, 95% CI = 1.10–1.16, *P* < .001; Table S4, Supplemental Digital Content), with distinct threshold-dependent effects. Below the RAR cutoff (<4.26), mortality risk increased dramatically (HR = 2.49, 95% CI = 2.18–2.84, *P* < .001; Table S5, Supplemental Digital Content), markedly exceeding the low-range effect of RDW (HR = 1.34, 95% CI = 1.28–1.41, *P* < .001). Notably, the RAR maintained prognostic value even above its threshold (HR = 1.32, 95% CI = 1.06–1.65, *P* = .012), whereas the RDW lost significance (*P* > .05) above 15.97. The biphasic dynamic threshold of the RAR increased the sensitivity to acute pathological processes (*P* < .001 in the low range) and exhibited superior multidimensional stratification capacity compared with the unidirectional predictive pattern of the RDW, which was confined to lower values (interaction *P* < .05). CKD patients with RAR > 4.26 (Fig. 4A) or RDW > 15.97 (Fig. 4B) exhibited significantly poorer survival outcomes.

### 3.6. Mediation analysis of the NLR and the SII

On the basis of the mediation analysis, RAR influences all-cause mortality primarily through the NLR rather than the SII. For the SII pathway, RAR→SII showed a significant association (β = 140.08, 95% CI = 97.11–183.06, *P* < .001), but the SII→ mortality path had minimal clinical relevance (β = 0.01, 95% CI = 0.00–0.00, *P* < .001), with a weak indirect effect (β = −2.10, 95% CI = −3.40 to −0.78, *P* < .001) mediating only 5.27% of the total effect. In contrast, for the NLR pathway, both RAR→NLR (β = 0.49, 95% CI = 0.36–0.62, *P* < .001) and NLR→ mortality (β = 0.13, 95% CI = 0.10–0.16, *P* < .001) demonstrated robust associations, yielding a stronger indirect effect (β = −3.18, 95% CI = −4.32 to −1.77, *P* < .001) and a higher mediation proportion (7.89%). The greater effect size and biological plausibility of the NLR confirmed its dominance over the SII (Tables S6,S7,S8,S9,S10,S11,S12,S13,S14 and S15, Supplemental Digital Content).

### 3.7. Subgroup analyses of the association between RAR and all-cause mortality

**Figure 5.**
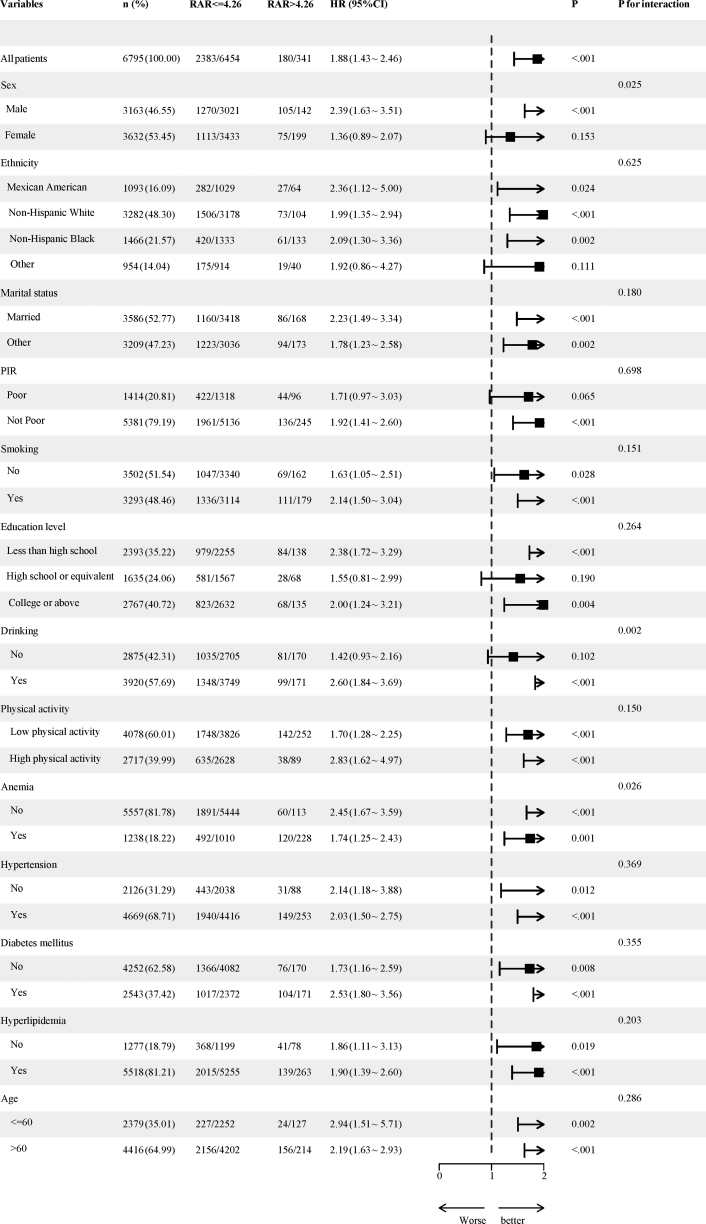
Subgroup analysis forest plot. CI = confidence interval, OR = odds ratio, PIR = poverty-to-income ratio, RAR = red cell distribution width-to-albumin ratio.

We investigated the associations between RAR levels and CKD all-cause mortality through subgroup analyses stratified by sex, alcohol consumption, and anemia (Table S16, Supplemental Digital Content). The analysis revealed significant subgroup heterogeneity in the association of RAR with all-cause mortality in CKD patients. Specifically, this association was statistically significant in males (HR = 2.39, *P* < .001) but not in females (HR = 1.36, *P* = .153). Significant associations were detected in alcohol consumers (HR = 2.60, *P* < .001) and nonanemic patients (HR = 2.45, *P* < .001), whereas the effect sizes were attenuated in nondrinkers (HR = 1.42, *P* = .102) and anemic patients (HR = 1.74, *P* = .001). Notably, significant interaction effects were detected for sex (*P* interaction = .025), drinking status (*P* interaction = .002), and anemia status (*P* interaction = .026), suggesting that these factors may modulate the impact of RAR on renal outcomes. In contrast, no significant interactions were detected in the other subgroups, including ethnicity (*P* interaction = .625), marital status (*P* interaction = .180), and hypertension (*P* interaction = .369; all *P* interaction > .05; Fig. 5).

## 4. Discussion

The present study establishes RAR as a novel integrative biomarker demonstrating robust prognostic utility for all-cause mortality in CKD patients. By combining erythrocyte heterogeneity (reflected by RDW) and systemic inflammation–nutritional imbalance (reflected by albumin),^[23]^ RAR uniquely encapsulates the complex interplay of pathological mechanisms driving CKD progression. These mechanisms encompass chronic inflammation, oxidative stress, anemia, and metabolic dysregulation, providing a more holistic reflection of cumulative disease burden than isolated parameters. Findings derived from a nationally representative cohort with extended follow-up demonstrate RAR’s superior predictive capability over traditional biomarkers.

Its superiority stems from the synergistic integration of complementary biological pathways – erythrocyte instability and hypoalbuminemia – which collectively signify the escalating burden of inflammation, malnutrition, and oxidative damage inherent in CKD pathogenesis. The relationship between RAR and mortality is notably nonlinear, exhibiting a critical threshold beyond which mortality risk rises substantially. This contrasts with isolated biomarkers, such as RDW, which lose discriminatory power beyond a certain point, underscoring RAR’s unique capacity to capture progressive pathological insults masked by single-parameter assessments.

CKD progression involves a self-perpetuating cycle of chronic inflammation driven by key cytokines, which exacerbate renal fibrosis, anemia, and malnutrition.^[24]^ Hypoalbuminemia, a defining feature of CKD, reflects not merely nutritional deficiency but also the profound impact of systemic inflammation, where albumin synthesis is actively suppressed and its degradation accelerated. Concurrently, elevated RDW emerges from oxidative damage to erythrocytes, erythropoiesis dysfunction triggered by uremic toxins, and resistance to erythropoietin.^[25]^

The composite structure of RAR bridges these distinct pathways, offering an integrated view of disease severity. For instance, the combination of high RDW and low albumin signifies concurrent erythrocyte destabilization and severe inflammatory-nutritional compromise, while isolated RDW elevation may lack diagnostic specificity due to other potential causes. This multidimensionality underpins RAR’s enhanced discriminative performance for mortality risk compared to component markers alone and enables effective stratification of patients into distinct prognostic categories. The clear dose–dependent gradient in mortality risk observed across ascending RAR levels further supports its clinical utility. This potential is particularly significant in resource-limited settings lacking access to advanced biomarker testing.

Subgroup analyses revealed significant interactions between RAR and sociodemographic factors, underscoring the necessity for personalized risk assessment. Compared to females, males exhibited a disproportionately greater increase in mortality risk per unit rise in RAR. This disparity potentially originates from sex-specific differences in immune regulation, hormonal influences on erythropoiesis, or prevalent comorbid conditions.^[26]^ Differences in sex hormone profiles, known to modulate cytokine production, offer one plausible biological explanation for this finding. Similarly, alcohol users face heightened RAR-associated mortality risks,^[27]^ likely due to alcohol’s dual role in amplifying oxidative stress and impairing nutritional status. Intriguingly, patients without anemia demonstrated significant vulnerability when RAR was elevated, suggesting that the absence of overt anemia might mask underlying persistent inflammation and ongoing damage. Striking ethnic disparities were also observed, with racial minorities exhibiting the highest mortality risk associated with RAR.^[28]^

These findings resonate with well-documented broader racial inequities in CKD outcomes, often driven by complex socioeconomic factors, potential genetic predispositions, and differential healthcare access. The observed interactions highlight RAR’s potential to not only predict individual risk but also to uncover systemic health inequities, thereby informing strategies for more equitable resource allocation within CKD management programs.

Mechanistically, mediation analysis confirmed a central role for neutrophilic inflammation in the pathway linking elevated RAR to mortality. This pathway demonstrated greater importance than platelet-associated inflammatory indices. The prominence of neutrophil-dominated mechanisms aligns with experimental models where neutrophils actively contribute to renal injury.^[29]^ RAR’s stability across complex multivariable models, even after extensive adjustment for numerous clinical and demographic covariates, underscores its resilience to confounding influences. Its robustness, combined with the routine availability and low cost of its component tests (RDW and albumin), positions RAR as a pragmatic biomarker ideally suited for integration into diverse clinical settings.

While acknowledging the strengths of this study – a large representative cohort, extended follow-up, and rigorous adjustment – several limitations warrant consideration. As an observational study, causality between RAR and mortality cannot be definitively established; unmeasured confounders such as dietary patterns, genetic variations, or occult comorbidities could partially contribute to the observed relationships. Self-reported data on alcohol use and comorbidities, despite standardized collection protocols, introduce potential misclassification bias. Furthermore, the absence of serial RAR measurements precludes analysis of temporal trends and prevents assessment of how fluctuations might correlate with disease exacerbation or therapeutic response. The study population primarily involved non-dialysis CKD patients; validating RAR’s utility in advanced CKD and dialysis-dependent populations, where inflammation and malnutrition intensify, is a critical next step. Furthermore, although the study accounted for numerous covariates, unmeasured inflammatory conditions (e.g., subclinical infections, autoimmune disorders) or acute illnesses could independently impact RDW (e.g., via altered erythropoiesis) or albumin levels (e.g., via acute phase response or capillary leak) independently of CKD activity. Such transient or concomitant conditions might obscure the relationship between RAR and underlying CKD severity or contribute to biomarker variability unrelated to the long-term CKD pathomechanisms RAR aims to capture. Finally, while mediation analysis highlighted neutrophilic inflammation as a key pathway linking RAR to mortality, other unexplored mechanisms, such as gut dysbiosis or endothelial dysfunction, might also contribute meaningfully.

Clinical utility expansion: the identification of a critical RAR threshold offers a tangible pathway for clinical implementation. RAR > 4.26 provides a clear, actionable cutoff point for clinicians. Integration into clinical workflows could involve automatically flagging patients exceeding this value within electronic health records during routine care visits or laboratory reviews. These patients could then trigger standardized management pathways, such as prioritization for nephrology consultation, expedited nutritional assessment and intervention,^[30]^ enhanced monitoring for signs of progressive inflammation or hematologic derangement, consideration for more frequent follow-up visits, or evaluation for eligibility in trials of targeted anti-inflammatory therapies (e.g., IL-6 inhibitors).^[31]^

This simple binary stratification (>4.26 vs ≤4.26) is particularly valuable in time-constrained settings, offering an immediate signal to intensify management focus and mitigate risk. The interaction effects highlighted in subgroup analyses further support a precision medicine approach utilizing RAR. For example, heightened vigilance and potentially more aggressive inflammation control might be warranted for males with elevated RAR, while alcohol cessation counseling should be prioritized for affected users. From a public health perspective, RAR’s ability to quantify pronounced disparities in mortality risk among racial minorities serves as concrete evidence to support policy initiatives focused on reducing CKD-related health inequities. Efforts could include improving access to care, addressing social determinants of health, and implementing culturally tailored interventions for vulnerable populations. The routine nature of RAR’s components underscores its significant potential for widespread adoption and impact in optimizing CKD management across diverse healthcare landscapes.

## 5. Conclusion

This study identifies RAR as a superior prognostic biomarker for all-cause mortality in CKD patients, integrating erythrocyte instability, systemic inflammation, and malnutrition into a single metric. With a critical threshold of 4.26, RAR enables dynamic risk stratification, outperforming isolated RDWs and demonstrating significant interactions with sex, alcohol use, and ethnicity. Mechanistically, neutrophilic inflammation (via the NLR) mediates RAR mortality risk, emphasizing its role in CKD progression. Despite observational limitations, RAR’s robustness, cost-effectiveness, and ability to highlight health disparities advocate its integration into clinical practice. Future research should validate RAR in advanced CKD cohorts and explore its utility in guiding targeted therapies, ultimately advancing personalized CKD management.

## Acknowledgments

We would like to acknowledge the participants and investigators of the National Health and Nutrition Examination Survey. The content is solely the responsibility of the authors and does not necessarily represent the official views of the National Institutes of Health.

## Author contributions

**Data curation:** Zhengyang Zhu, Dong Wang.

**Formal analysis:** Zhengyang Zhu, Xiaowei Duan.

**Methodology:** Zhengyang Zhu, Dong Wang.

**Visualization:** Zhengyang Zhu.

**Writing – original draft:** Zhengyang Zhu, Kejun Ren, Lei Zhang, Dong Wang.

**Investigation:** Kejun Ren, Xulei Hu.

**Resources:** Kejun Ren, Hua Jin.

**Writing – review & editing:** Xiaowei Duan, Xulei Hu, Yong Lv, Dong Wang, Hua Jin, Lei Zhang.

**Conceptualization:** Hua Jin.

**Supervision:** Hua Jin.
































