# Supplementary material for: Red cell distribution width-to-albumin ratio and chronic kidney disease mortality in adults: A population-based NHANES 1999 to 2020 study
Source: Medicine (Baltimore). 2026 Jun 12;105(24):e44559. doi: 10.1097/MD.0000000000044559 (PMC13268450; doi:10.1097/MD.0000000000044559)
Supplement: Supplementary file 1 [file medi-105-e44559-s001.docx]

Table S1: Characteristic of RAR quartile.

| Variable | Total  (n = 40133) | Q1  (n=7437) | Q2  (n=8868) | Q3  (n=10724) | Q4  (n=13104) | Statistic | *P* |
| --- | --- | --- | --- | --- | --- | --- | --- |
|  |  |  |  |  |  |  |  |
| ALB, Mean (SE) | 4.33 (0.01) | 4.69 (0.01) | 4.42 (0.00) | 4.25 (0.00) | 3.98 (0.00) | F=11547.29 | <.001 |
| ALT, Mean (SE) | 25.77(0.16) | 27.85(0.33) | 26.52 (0.26) | 25.26 (0.21) | 23.50 (0.27) | F=126.36 | <.001 |
| AST, Mean (SE) | 25.47(0.11) | 25.99(0.20) | 25.52 (0.21) | 25.26 (0.18) | 25.12 (0.23) | F=10.21 | 0.002 |
| Ca, Mean (SE) | 9.44(0.01) | 9.61(0.02) | 9.48 (0.01) | 9.40 (0.01) | 9.28 (0.01) | F=522.69 | <.001 |
| HCO3, Mean(SE) | 24.71(0.06) | 24.82(0.10) | 24.76 (0.07) | 24.76 (0.07) | 24.51 (0.06) | F=10.29 | 0.002 |
| GGT, Mean (SE) | 27.88(0.26) | 27.78(0.42) | 26.93 (0.48) | 28.17 (0.57) | 28.63 (0.52) | F=3.21 | 0.076 |
| Glu, Mean (SE) | 97.45 (0.23) | 91.63 (0.28) | 94.89 (0.35) | 99.20 (0.42) | 103.98 (0.41) | F=920.68 | <.001 |
| TP, Mean (SE) | 71.96 (0.10) | 74.73 (0.14) | 72.33 (0.10) | 70.96 (0.10) | 69.86 (0.10) | F=1222.29 | <.001 |
| TG, Mean (SE) | 146.84 (1.13) | 140.89 (2.08) | 148.93 (2.00) | 152.25 (1.86) | 145.19 (1.47) | F=5.45 | 0.021 |
| UA, Mean (SE) | 5.38 (0.01) | 5.49 (0.02) | 5.38 (0.02) | 5.36 (0.02) | 5.28 (0.02) | F=73.36 | <.001 |
| SCr, Mean (SE) | 0.85 (0.00) | 0.84 (0.00) | 0.84 (0.00) | 0.85 (0.00) | 0.88 (0.01) | F=29.60 | <.001 |
| Na, Mean (SE) | 139.19 (0.06) | 139.40 (0.11) | 139.23 (0.06) | 139.19 (0.06) | 138.94 (0.06) | F=23.23 | <.001 |
| K, Mean (SE) | 4.01 (0.01) | 4.02 (0.01) | 4.00 (0.01) | 4.00 (0.01) | 4.01 (0.01) | F=1.71 | 0.194 |
| Cl, Mean (SE) | 103.50 (0.08) | 102.75 (0.15) | 103.46 (0.09) | 103.80 (0.07) | 103.98 (0.08) | F=88.99 | <.001 |
| LymP, Mean (SE) | 29.97 (0.08) | 30.49 (0.11) | 30.43 (0.11) | 30.08 (0.12) | 28.88 (0.14) | F=111.52 | <.001 |
| MonP, Mean (SE) | 7.97 (0.02) | 8.01 (0.05) | 7.95 (0.03) | 8.00 (0.04) | 7.91 (0.04) | F=2.35 | 0.128 |
| EoP, Mean (SE) | 2.82 (0.01) | 2.73 (0.03) | 2.81 (0.03) | 2.89 (0.03) | 2.83 (0.02) | F=11.35 | 0.001 |
| BaP, Mean (SE) | 0.70 (0.01) | 0.67 (0.01) | 0.69 (0.01) | 0.71 (0.01) | 0.74 (0.01) | F=39.69 | <.001 |
| Lym, Mean (SE) | 2.13 (0.01) | 2.10 (0.01) | 2.12 (0.01) | 2.15 (0.01) | 2.15 (0.01) | F=7.40 | 0.007 |
| Mon, Mean (SE) | 0.56 (0.00) | 0.55 (0.00) | 0.55 (0.00) | 0.57 (0.00) | 0.58 (0.00) | F=44.85 | <.001 |
| Segne, Mean (SE) | 4.34 (0.02) | 4.17 (0.03) | 4.22 (0.03) | 4.32 (0.03) | 4.63 (0.03) | F=166.80 | <.001 |
| Eo, Mean (SE) | 0.20 (0.00) | 0.19 (0.00) | 0.20 (0.00) | 0.21 (0.00) | 0.21 (0.00) | F=49.02 | <.001 |
| Ba, Mean (SE) | 0.04(0.00) | 0.04(0.00) | 0.04 (0.00) | 0.04 (0.00) | 0.05 (0.00) | F=123.56 | <.001 |
| RBC, Mean (SE) | 4.70(0.01) | 4.83(0.01) | 4.73(0.01) | 4.69 (0.01) | 4.56 (0.01) | F=404.09 | <.001 |
| Hg, Mean (SE) | 14.32 (0.02) | 15.02 (0.04) | 14.61 (0.03) | 14.32 (0.03) | 13.36 (0.03) | F=1528.79 | <.001 |
| Hem, Mean (SE) | 42.15 (0.07) | 43.91 (0.10) | 42.80 (0.08) | 42.13 (0.08) | 39.77 (0.08) | F=1264.44 | <.001 |
| MCH, Mean (SE) | 89.83 (0.07) | 91.05 (0.11) | 90.60 (0.08) | 90.11 (0.09) | 87.57 (0.12) | F=451.18 | <.001 |
| MCHC, Mean(SE) | 30.53 (0.03) | 31.14 (0.04) | 30.92 (0.03) | 30.63 (0.04) | 29.42 (0.06) | F=705.74 | <.001 |
| RDW, Mean (SE) | 12.92 (0.01) | 12.09 (0.01) | 12.51 (0.01) | 12.94 (0.01) | 14.12 (0.02) | F=10679.00 | <.001 |
| MPV, Mean (SE) | 8.18(0.01) | 8.14(0.02) | 8.15(0.02) | 8.18(0.02) | 8.24 (0.02) | F=23.99 | <.001 |
| HDL, Mean (SE) | 53.00 (0.22) | 52.44 (0.33) | 52.71 (0.32) | 53.19 (0.31) | 53.64 (0.24) | F=12.27 | <.001 |
| Ualb, Mean (SE) | 32.03 (1.47) | 16.65 (1.08) | 20.69 (2.15) | 25.16 (1.74) | 65.50 (4.76) | F=114.36 | <.001 |
| BMI, Mean (SE) | 28.48 (0.07) | 25.92 (0.09) | 27.60 (0.10) | 28.94 (0.10) | 31.44 (0.11) | F=1884.85 | <.001 |
| Age, Mean (SE) | 45.55 (0.20) | 38.07 (0.31) | 44.30 (0.26) | 48.73 (0.28) | 50.99 (0.28) | F=1446.49 | <.001 |
| UACR, Mean (SE) | 30.52 (1.56) | 13.25 (0.78) | 19.24 (2.61) | 22.85 (1.63) | 66.64 (4.65) | F=143.65 | <.001 |
| EGFR, Mean (SE) | 103.91 (0.27) | 115.08 (0.48) | 106.26 (0.40) | 100.02 (0.36) | 94.46 (0.41) | F=1690.25 | <.001 |
| SII, Mean (SE) | 565.39 (3.36) | 542.31 (4.71) | 538.66 (5.51) | 548.27 (4.83) | 632.16 (5.83) | F=148.00 | <.001 |
| NLR, Mean (SE) | 2.22(0.01) | 2.13(0.02) | 2.13(0.01) | 2.19 (0.02) | 2.43 (0.02) | F=178.90 | <.001 |
| Sex, n(%) |  |  |  |  |  | χ²=2200.46 | <.001 |
| Male | 19447 (48.37) | 4924 (63.85) | 4943 (52.47) | 5118 (45.90) | 4462 (31.45) |  |  |
| Female | 20686 (51.63) | 2513 (36.15) | 3925 (47.53) | 5606 (54.10) | 8642 (68.55) |  |  |
| Ethnicity, n(%) |  |  |  |  |  | χ²=1527.07 | <.001 |
| Mexican American | 7531 (8.15) | 1657 (8.01) | 1816 (8.31) | 1966 (8.22) | 2092 (8.08) |  |  |
| Non-Hispanic White | 17452 (68.88) | 3785 (75.23) | 4141 (71.95) | 4718 (68.82) | 4808 (59.58) |  |  |
| Non-Hispanic Black | 8253(10.72) | 744 (4.54) | 1291(7.15) | 2097(10.82) | 4121(20.27) |  |  |
| Other | 6897 (12.25) | 1251 (12.22) | 1620 (12.59) | 1943 (12.14) | 2083 (12.07) |  |  |
| Marital status, n(%) |  |  |  |  |  | χ²=159.24 | <.001 |
| Married | 22600 (60.57) | 3930 (57.13) | 5304 (64.43) | 6308 (62.73) | 7058 (57.99) |  |  |
| Other (widowed, divorced, separated, never married, living with a partner) | 17533 (39.43) | 3507 (42.87) | 3564 (35.57) | 4416 (37.27) | 6046 (42.01) |  |  |
| PIR, n(%) |  |  |  |  |  | χ²=183.68 | <.001 |
| Poor | 8159 (13.67) | 1373 (11.77) | 1643 (11.86) | 2093 (13.50) | 3050 (17.51) |  |  |
| Not Poor | 31974 (86.33) | 6064 (88.23) | 7225 (88.14) | 8631 (86.50) | 10054 (82.49) |  |  |
| Smoking, n(%) |  |  |  |  |  | χ²=29.86 | 0.008 |
| No | 23056 (54.90) | 4560 (57.18) | 5177 (54.67) | 6016 (53.69) | 7303 (54.09) |  |  |
| Yes | 17077 (45.10) | 2877 (42.82) | 3691 (45.33) | 4708 (46.31) | 5801 (45.91) |  |  |
| Education level, n(%) |  |  |  |  |  | χ²=172.99 | <.001 |
| Less than high school | 11336 (18.84) | 1850 (15.88) | 2416 (17.93) | 3093 (19.45) | 3977 (22.04) |  |  |
| high school or equivalent | 9486 (23.87) | 1768 (22.87) | 2081 (24.74) | 2459 (23.28) | 3178 (24.61) |  |  |
| college or above | 19311 (57.29) | 3819 (61.25) | 4371 (57.33) | 5172 (57.26) | 5949 (53.35) |  |  |
| Drinking, n(%) |  |  |  |  |  | χ²=335.43 | <.001 |
| No | 15375 (31.23) | 2633 (27.05) | 3141 (28.46) | 3947 (31.27) | 5654 (38.08) |  |  |
| Yes | 24758 (68.77) | 4804 (72.95) | 5727 (71.54) | 6777 (68.73) | 7450 (61.92) |  |  |
| Physical activity, n(%) |  |  |  |  |  | χ²=495.74 | <.001 |
| Low physical activity | 18538 (43.67) | 2844 (38.01) | 3602 (40.05) | 4949 (44.13) | 7143 (52.41) |  |  |
| High physical activity | 21595 (56.33) | 4593 (61.99) | 5266 (59.95) | 5775 (55.87) | 5961 (47.59) |  |  |
| Anemia, n(%) |  |  |  |  |  | χ²=3915.43 | <.001 |
| No | 36377 (93.73) | 7353 (99.21) | 8661 (98.34) | 10214 (96.70) | 10149 (80.72) |  |  |
| Yes | 3756(6.27) | 84 (0.79) | 207 (1.66) | 510 (3.30) | 2955(19.28) |  |  |
| Hypertension, n(%) |  |  |  |  |  | χ²=1326.21 | <.001 |
| No | 24776 (65.81) | 5678 (77.14) | 6014 (70.23) | 6351 (61.86) | 6733 (54.21) |  |  |
| Yes | 15357 (34.19) | 1759 (22.86) | 2854 (29.77) | 4373 (38.14) | 6371 (45.79) |  |  |
| **Diabetes mellitus**, n(%) |  |  |  |  |  | χ²=1277.62 | <.001 |
| No | 33723 (88.26) | 6935 (95.05) | 7847 (91.68) | 8949 (86.66) | 9992 (79.78) |  |  |
| Yes | 6410(11.74) | 502 (4.95) | 1021(8.32) | 1775(13.34) | 3112(20.22) |  |  |
| Hyperlipidemia, n(%) |  |  |  |  |  | χ²=257.86 | <.001 |
| No | 11960 (29.03) | 2820 (34.75) | 2774 (29.67) | 2968 (26.22) | 3398 (25.58) |  |  |
| Yes | 28173 (70.97) | 4617 (65.25) | 6094 (70.33) | 7756 (73.78) | 9706 (74.42) |  |  |
| CKD, n(%) |  |  |  |  |  | χ²=1176.03 | <.001 |
| No | 33338 (86.73) | 6807 (92.96) | 7860 (90.83) | 8907 (85.32) | 9764 (77.92) |  |  |
| Yes | 6795(13.27) | 630 (7.04) | 1008(9.17) | 1817(14.68) | 3340(22.08) |  |  |
| All estimates accounted for complex survey designs. RAR, red cell distribution width-to-albumin ratio; CKD, chronic kidney disease; UACR, urinary albumin‒creatinine ratio; EGFR, estimated glomerular filtration rate; NLR, neutrophil-to-lymphocyte ratio; SII, systemic immune-inflammatory index; Na, sodium; K, potassium; Cl, chloride; HCO₃⁻, bicarbonate; Ca, total calcium; Scr, creatinine; ALB, albumin; TP, total protein; Glu, glucose; TG, triglycerides; HDL, high-density lipoprotein; UA, uric acid; ALT, alanine transaminase; AST, aspartate transaminase; GGT, gamma-glutamyl transferase; BAP, basophil percentage; LymP, lymphocyte percentage; MonP, monocyte percentage; EoP, eosinophil percentage; Lym, absolute lymphocyte count; Mon, monocyte count; Eo eosinophil count; Senge, segmented neutrophil count; Ba, basophil count; RBC, red blood cell count; Hg, hemoglobin; Hem, hematocrit; MCH, mean corpuscular hemoglobin; MCHC, mean corpuscular hemoglobin concentration; RDW, red cell distribution width; MPV, mean platelet volume; MCV, mean corpuscular volume; PIR, poverty income ratio; BMI, body mass index; t: t test, χ²: Chi-square test. | | | | | | | |
