# Supplementary material for: Red cell distribution width-to-albumin ratio and chronic kidney disease mortality in adults: A population-based NHANES 1999 to 2020 study
Source: Medicine (Baltimore). 2026 Jun 12;105(24):e44559. doi: 10.1097/MD.0000000000044559 (PMC13268450; doi:10.1097/MD.0000000000044559)
Supplement: Supplementary file 2 [file medi-105-e44559-s002.docx]

Table S2: Sensitivity analysis stratified by gender

| Sex | | | Variables | CKD | | | | | | | |
| --- | --- | --- | --- | --- | --- | --- | --- | --- | --- | --- | --- |
|  |  |  |  | HR (95%CI) | *P* | HR (95%CI) | *P* | HR (95%CI) | *P* | HR (95%CI) | *P* |
| Male | | | RAR | 2.29 (1.96 - 2.69) | <.001 | 1.90 (1.62 - 2.23) | <.001 | 1.76 (1.51 - 2.05) | <.001 | 1.98 (1.66 - 2.37) | <.001 |
|  |  |  | Q1 | 1.00 (Reference) |  | 1.00 (Reference) |  | 1.00 (Reference) |  | 1.00 (Reference) |  |
|  |  |  | Q2 | 1.77 (1.41 - 2.23) | <.001 | 1.20 (0.94 - 1.53) | 0.144 | 1.22 (0.92 - 1.61) | 0.162 | 1.28 (0.97 - 1.69) | 0.084 |
|  |  |  | Q3 | 2.78 (2.12 - 3.65) | <.001 | 1.52 (1.17 - 1.98) | 0.002 | 1.48 (1.12 - 1.96) | 0.007 | 1.69 (1.26 - 2.26) | <.001 |
|  |  |  | Q4 | 4.84 (3.85 - 6.09) | <.001 | 2.39 (1.86 - 3.08) | <.001 | 2.10 (1.59 - 2.77) | <.001 | 2.30 (1.67 - 3.17) | <.001 |
| Female | | | RAR | 1.66 (1.51 - 1.84) | <.001 | 1.92 (1.69 - 2.18) | <.001 | 1.77 (1.53 - 2.05) | <.001 | 1.90 (1.60 - 2.25) | <.001 |
|  |  |  | 1 | 1.00 (Reference) |  | 1.00 (Reference) |  | 1.00 (Reference) |  | 1.00 (Reference) |  |
|  |  |  | 2 | 1.49 (1.17 - 1.88) | <.001 | 1.13 (0.91 - 1.39) | 0.266 | 1.06 (0.87 - 1.31) | 0.552 | 1.15 (0.90 - 1.47) | 0.252 |
|  |  |  | 3 | 2.03 (1.64 - 2.51) | <.001 | 1.36 (1.09 - 1.70) | 0.006 | 1.24 (1.01 - 1.53) | 0.050 | 1.43 (1.12 - 1.82) | 0.004 |
|  |  |  | 4 | 2.98 (2.49 - 3.57) | <.001 | 2.52 (2.09 - 3.05) | <.001 | 2.09 (1.69 - 2.58) | <.001 | 2.42 (1.84 - 3.16) | <.001 |

### RAR, red cell distribution width-to-albumin ratio; CKD, chronic kidney disease; HR, hazard ratio; CI, confidence interval.
