# Supplementary material for: Red cell distribution width-to-albumin ratio and chronic kidney disease mortality in adults: A population-based NHANES 1999 to 2020 study
Source: Medicine (Baltimore). 2026 Jun 12;105(24):e44559. doi: 10.1097/MD.0000000000044559 (PMC13268450; doi:10.1097/MD.0000000000044559)
Supplement: Supplementary file 3 [file medi-105-e44559-s003.docx]

Table S3: Baseline characteristics of CKD patients stratified by survival status​

| Variable | Total  (n = 6795) | CKD patients alive  (n=4232) | CKD patients dead  (n=2563) | Statistic | *P* |
| --- | --- | --- | --- | --- | --- |
|  |  |  |  |  |  |
| ALB, Mean (SE) | 4.22(0.01) | 4.26(0.01) | 4.14(0.01) | t=-10.76 | <.001 |
| ALT, Mean (SE) | 24.59(0.33) | 25.43 (0.38) | 22.91(0.63) | t=-3.40 | <.001 |
| AST, Mean (SE) | 26.46 (0.30) | 26.21(0.31) | 26.97(0.64) | t=1.05 | 0.295 |
| Ca, Mean (SE) | 9.47(0.01) | 9.47(0.01) | 9.47(0.02) | t=0.48 | 0.634 |
| HCO3, Mean (SE) | 24.75(0.07) | 24.64(0.07) | 24.98(0.10) | t=3.99 | <.001 |
| GGT, Mean (SE) | 33.47(1.04) | 30.51(0.79) | 39.38(2.66) | t=3.21 | 0.002 |
| Glu, Mean (SE) | 113.25(0.75) | 110.33(0.89) | 119.10(1.36) | t=5.37 | <.001 |
| TP, Mean (SE) | 71.84(0.14) | 71.88 (0.14) | 71.76 (0.21) | t=-0.65 | 0.517 |
| TG, Mean (SE) | 168.52(2.36) | 169.00(2.97) | 167.57(3.43) | t=-0.33 | 0.745 |
| UA, Mean (SE) | 5.92(0.03) | 5.74(0.04) | 6.26(0.04) | t=8.82 | <.001 |
| SCr, Mean (SE) | 1.08(0.01) | 1.00(0.01) | 1.23(0.02) | t=9.42 | <.001 |
| Na, Mean (SE) | 139.18 (0.07) | 139.18(0.07) | 139.19(0.11) | t=0.09 | 0.926 |
| K, Mean (SE) | 4.08(0.01) | 4.03(0.01) | 4.19(0.01) | t=10.34 | <.001 |
| Cl, Mean (SE) | 103.10(0.10) | 103.42(0.10) | 102.46(0.14) | t=-7.74 | <.001 |
| LymP, Mean (SE) | 27.85(0.16) | 28.95(0.19) | 25.65(0.20) | t=-13.04 | <.001 |
| MonP, Mean (SE) | 8.06(0.05) | 7.85(0.05) | 8.50(0.08) | t=7.87 | <.001 |
| EoP, Mean (SE) | 2.91(0.04) | 2.83(0.04) | 3.07(0.06) | t=3.18 | 0.002 |
| BaP, Mean (SE) | 0.72(0.01) | 0.73(0.01) | 0.69(0.01) | t=-2.89 | 0.005 |
| Lym, Mean (SE) | 2.07(0.02) | 2.12 (0.02) | 1.97(0.04) | t=-3.41 | <.001 |
| Mon, Mean (SE) | 0.59(0.00) | 0.57(0.00) | 0.63(0.01) | t=7.82 | <.001 |
| Segne, Mean (SE) | 4.62(0.04) | 4.53(0.04) | 4.80(0.06) | t=3.82 | <.001 |
| Eo, Mean (SE) | 0.21(0.00) | 0.21(0.00) | 0.23(0.00) | t=3.66 | <.001 |
| Ba, Mean (SE) | 0.05(0.00) | 0.05(0.00) | 0.05(0.00) | t=-1.69 | 0.094 |
| RBC, Mean (SE) | 4.57(0.01) | 4.63(0.01) | 4.47(0.01) | t=-9.78 | <.001 |
| Hg, Mean (SE) | 13.94 (0.03) | 14.03(0.04) | 13.77(0.05) | t=-5.79 | <.001 |
| Hem, Mean (SE) | 41.17(0.09) | 41.38(0.10) | 40.77(0.12) | t=-4.77 | <.001 |
| MCH, Mean (SE) | 90.29(0.13) | 89.67(0.16) | 91.53(0.17) | t=8.96 | <.001 |
| MCHC, Mean (SE) | 30.58(0.06) | 30.41(0.06) | 30.92(0.07) | t=6.66 | <.001 |
| RDW, Mean (SE) | 13.36(0.02) | 13.24(0.03) | 13.58(0.04) | t=8.05 | <.001 |
| MPV, Mean (SE) | 8.21(0.02) | 8.24(0.03) | 8.15(0.03) | t=-3.10 | 0.002 |
| HDL, Mean (SE) | 52.70(0.36) | 52.69(0.43) | 52.71(0.56) | t=0.04 | 0.971 |
| Ualb, Mean (SE) | 180.64(10.71) | 151.76(8.69) | 238.4(25.25) | t=3.34 | 0.001 |
| BMI, Mean (SE) | 29.55 (0.14) | 29.69(0.16) | 29.27(0.22) | t=-1.67 | 0.097 |
| Age, Mean (SE) | 59.12(0.39) | 52.86(0.47) | 71.64(0.35) | t=33.51 | <.001 |
| UACR, Mean (SE) | 180.56(11.30) | 143.73 (10.22) | 254.2(23.62) | t=4.56 | <.001 |
| EGFR, Mean (SE) | 79.94(0.60) | 86.93(0.80) | 65.96(0.70) | t=-18.78 | <.001 |
| RAR, Mean (SE) | 3.19(0.01) | 3.13(0.01) | 3.31(0.01) | t=11.55 | <.001 |
| SII, Mean (SE) | 634.79(9.05) | 586.83(6.69) | 730.8(20.30) | t=7.01 | <.001 |
| NLR, Mean (SE) | 2.55(0.03) | 2.34(0.02) | 2.95(0.05) | t=10.43 | <.001 |
| Sex, n(%) |  |  |  | χ²=26.30 | <.001 |
| Male | 3163(42.23) | 1788(40.06) | 1375(46.58) |  |  |
| Female | 3632(57.77) | 2444(59.94) | 1188(53.42) |  |  |
| Ethnicity, n(%) |  |  |  | χ²=153.47 | <.001 |
| Mexican American | 1093(7.04) | 784(8.64) | 309(3.85) |  |  |
| Non-Hispanic White | 3282(69.40) | 1703(64.87) | 1579(78.45) |  |  |
| Non-Hispanic Black | 1466(11.96) | 985(12.65) | 481(10.58) |  |  |
| Other | 954(11.60) | 760(13.84) | 194(7.12) |  |  |
| Marital status, n(%) |  |  |  | χ²=49.04 | <.001 |
| Married | 3586(55.97) | 2340(58.95) | 1246(50.00) |  |  |
| Other (widowed, divorced, separated, never married, living with a partner) | 3209(44.03) | 1892(41.05) | 1317(50.00) |  |  |
| PIR, n(%) |  |  |  | χ²=10.05 | 0.014 |
| Poor | 1414(16.26) | 948(17.26) | 466(14.25) |  |  |
| Not Poor | 5381(83.74) | 3284(82.74) | 2097(85.75) |  |  |
| Smoking, n(%) |  |  |  | χ²=58.68 | <.001 |
| No | 3502(51.16) | 2386(54.44) | 1116(44.59) |  |  |
| Yes | 3293(48.84) | 1846(45.56) | 1447(55.41) |  |  |
| Education level, n(%) |  |  |  | χ²=159.54 | <.001 |
| Less than high school | 2393(25.67) | 1330(21.37) | 1063(34.27) |  |  |
| High school or equivalent | 1635(25.52) | 1026(25.14) | 609(26.29) |  |  |
| College or above | 2767(48.80) | 1876(53.48) | 891(39.44) |  |  |
| Drinking, n(%) |  |  |  | χ²=49.16 | <.001 |
| No | 2875(38.48) | 1759(35.55) | 1116(44.33) |  |  |
| Yes | 3920(61.52) | 2473(64.45) | 1447(55.67) |  |  |
| Physical activity, n(%) |  |  |  | χ²=388.69 | <.001 |
| Low physical activity | 4078(56.60) | 2188(48.22) | 1890(73.37) |  |  |
| High physical activity | 2717(43.40) | 2044(51.78) | 673(26.63) |  |  |
| Anemia, n(%) |  |  |  | χ²=134.85 | <.001 |
| No | 5557(86.56) | 3606(89.95) | 1951(79.76) |  |  |
| Yes | 1238(13.44) | 626(10.05) | 612(20.24) |  |  |
| Hypertension, n(%) |  |  |  | χ²=463.18 | <.001 |
| No | 2126(36.77) | 1652(45.67) | 474(18.96) |  |  |
| Yes | 4669(63.23) | 2580(54.33) | 2089(81.04) |  |  |
| **Diabetes mellitus**, n(%) |  |  |  | χ²=138.41 | <.001 |
| No | 4252(68.94) | 2810(73.61) | 1442(59.59) |  |  |
| Yes | 2543(31.06) | 1422(26.39) | 1121(40.41) |  |  |
| Hyperlipidemia, n(%) |  |  |  | χ²=38.87 | <.001 |
| No | 1277(18.01) | 868(20.06) | 409(13.90) |  |  |
| Yes | 5518(81.99) | 3364(79.94) | 2154(86.10) |  |  |
| All estimates accounted for complex survey designs. RAR, red cell distribution width-to-albumin ratio; CKD, chronic kidney disease; UACR, urinary albumin‒creatinine ratio; EGFR, estimated glomerular filtration rate; NLR, neutrophil-to-lymphocyte ratio; SII, systemic immune-inflammatory index; Na, sodium; K, potassium; Cl, chloride; HCO₃⁻, bicarbonate; Ca, total calcium; Scr, creatinine; ALB, albumin; TP, total protein; Glu, glucose; TG, triglycerides; HDL, high-density lipoprotein; UA, uric acid; ALT, alanine transaminase; AST, aspartate transaminase; GGT, gamma-glutamyl transferase; BAP, basophil percentage; LymP, lymphocyte percentage; MonP, monocyte percentage; EoP, eosinophil percentage; Lym, absolute lymphocyte count; Mon, monocyte count; Eo eosinophil count; Senge, segmented neutrophil count; Ba, basophil count; RBC, red blood cell count; Hg, hemoglobin; Hem, hematocrit; MCH, mean corpuscular hemoglobin; MCHC, mean corpuscular hemoglobin concentration; RDW, red cell distribution width; MPV, mean platelet volume; MCV, mean corpuscular volume; PIR, poverty income ratio; BMI, body mass index; t: t test, χ²: Chi-square test. | | | | | |
