# Supplementary material for: Red cell distribution width-to-albumin ratio and chronic kidney disease mortality in adults: A population-based NHANES 1999 to 2020 study
Source: Medicine (Baltimore). 2026 Jun 12;105(24):e44559. doi: 10.1097/MD.0000000000044559 (PMC13268450; doi:10.1097/MD.0000000000044559)
Supplement: Supplementary file 4 [file medi-105-e44559-s004.docx]

**Table S4**. Threshold Analysis Revealing Threshold Effects of RDW

| Outcome | effect | *P* |
| --- | --- | --- |
|  |  |  |
| Model 1 Fitting model by standard linear regression | 1.13 (1.10 - 1.16) | <.001 |
| Model 2 Fitting model by two-piecewise linear regression |  |  |
| Inflection point | 15.965 |  |
| <15.965 | 1.34 (1.28 - 1.41) | <.001 |
| ≥15.965 | 1.07 (1.00 - 1.15) | 0.059 |
| P for likelihood test |  | <.001 |
