# Supplementary material for: Red cell distribution width-to-albumin ratio and chronic kidney disease mortality in adults: A population-based NHANES 1999 to 2020 study
Source: Medicine (Baltimore). 2026 Jun 12;105(24):e44559. doi: 10.1097/MD.0000000000044559 (PMC13268450; doi:10.1097/MD.0000000000044559)
Supplement: Supplementary file 5 [file medi-105-e44559-s005.docx]

**Table S5**. Threshold Analysis Revealing Threshold Effects of RAR​

| Outcome | effect | *P* |
| --- | --- | --- |
|  |  |  |
| Model 1 Fitting model by standard linear regression | 1.70 (1.58 - 1.82) | <.001 |
| Model 2 Fitting model by two-piecewise linear regression |  |  |
| Inflection point | 4.263 |  |
| <=4.263 | 2.49 (2.18 - 2.84) | <.001 |
| >4.263 | 1.32 (1.06 - 1.65) | 0.013 |
| P for likelihood test |  | <.001 |
