# Supplementary material for: Red cell distribution width-to-albumin ratio and chronic kidney disease mortality in adults: A population-based NHANES 1999 to 2020 study
Source: Medicine (Baltimore). 2026 Jun 12;105(24):e44559. doi: 10.1097/MD.0000000000044559 (PMC13268450; doi:10.1097/MD.0000000000044559)
Supplement: Supplementary file 6 [file medi-105-e44559-s006.docx]

Table S6. Regression analysis of exposure and mediator (NLR model)

| Variables | β | SE | t | P | β (95%CI) |
| --- | --- | --- | --- | --- | --- |
| Intercept | 9.68 | 2.22 | 4.36 | <.001 | 9.68 (5.33 ~ 14.02) |
| RAR | 0.49 | 0.07 | 7.38 | <.001 | 0.49 (0.36 ~ 0.62) |

### RAR, red cell distribution width-to-albumin ratio; CI, confidence interval.
