# Supplementary material for: Red cell distribution width-to-albumin ratio and chronic kidney disease mortality in adults: A population-based NHANES 1999 to 2020 study
Source: Medicine (Baltimore). 2026 Jun 12;105(24):e44559. doi: 10.1097/MD.0000000000044559 (PMC13268450; doi:10.1097/MD.0000000000044559)
Supplement: Supplementary file 7 [file medi-105-e44559-s007.docx]

**Table S16.** Subgroup analyses of the association between RAR and all-cause mortality

| Variables | n (%) | RAR<=4.26 | RAR>4.26 | HR (95%CI) | *P* | P for interaction |
| --- | --- | --- | --- | --- | --- | --- |
|  |  |  |  |  |  |  |
| All patients | 6795 (100.00) | 2383/6454 | 180/341 | 1.88 (1.43 ~ 2.46) | <.001 |  |
| Sex, n(%) |  |  |  |  |  | 0.025 |
| Male | 3163 (46.55) | 1270/3021 | 105/142 | 2.39 (1.63 ~ 3.51) | <.001 |  |
| Female | 3632 (53.45) | 1113/3433 | 75/199 | 1.36 (0.89 ~ 2.07) | 0.153 |  |
| Ethnicity, n(%) |  |  |  |  |  | 0.625 |
| Mexican American | 1093 (16.09) | 282/1029 | 27/64 | 2.36 (1.12 ~ 5.00) | 0.024 |  |
| Non-Hispanic White | 3282 (48.30) | 1506/3178 | 73/104 | 1.99 (1.35 ~ 2.94) | <.001 |  |
| Non-Hispanic Black | 1466 (21.57) | 420/1333 | 61/133 | 2.09 (1.30 ~ 3.36) | 0.002 |  |
| Other | 954 (14.04) | 175/914 | 19/40 | 1.92 (0.86 ~ 4.27) | 0.111 |  |
| Marital status, n(%) |  |  |  |  |  | 0.180 |
| Married | 3586 (52.77) | 1160/3418 | 86/168 | 2.23 (1.49 ~ 3.34) | <.001 |  |
| Other (widowed, divorced, separated, never married, living with a partner) | 3209 (47.23) | 1223/3036 | 94/173 | 1.78 (1.23 ~ 2.58) | 0.002 |  |
| PIR, n(%) |  |  |  |  |  | 0.698 |
| Poor | 1414 (20.81) | 422/1318 | 44/96 | 1.71 (0.97 ~ 3.03) | 0.065 |  |
| Not Poor | 5381 (79.19) | 1961/5136 | 136/245 | 1.92 (1.41 ~ 2.60) | <.001 |  |
| Smoking, n(%) |  |  |  |  |  | 0.151 |
| No | 3502 (51.54) | 1047/3340 | 69/162 | 1.63 (1.05 ~ 2.51) | 0.028 |  |
| Yes | 3293 (48.46) | 1336/3114 | 111/179 | 2.14 (1.50 ~ 3.04) | <.001 |  |
| Education level, n(%) |  |  |  |  |  | 0.264 |
| Less than high school | 2393 (35.22) | 979/2255 | 84/138 | 2.38 (1.72 ~ 3.29) | <.001 |  |
| High school or equivalent | 1635 (24.06) | 581/1567 | 28/68 | 1.55 (0.81 ~ 2.99) | 0.190 |  |
| College or above | 2767 (40.72) | 823/2632 | 68/135 | 2.00 (1.24 ~ 3.21) | 0.004 |  |
| Drinking, n(%) |  |  |  |  |  | 0.002 |
| No | 2875 (42.31) | 1035/2705 | 81/170 | 1.42 (0.93 ~ 2.16) | 0.102 |  |
| Yes | 3920 (57.69) | 1348/3749 | 99/171 | 2.60 (1.84 ~ 3.69) | <.001 |  |
| Physical activity, n(%) |  |  |  |  |  | 0.150 |
| Low physical activity | 4078 (60.01) | 1748/3826 | 142/252 | 1.70 (1.28 ~ 2.25) | <.001 |  |
| High physical activity | 2717 (39.99) | 635/2628 | 38/89 | 2.83 (1.62 ~ 4.97) | <.001 |  |
| Anemia, n(%) |  |  |  |  |  | 0.026 |
| No | 5557 (81.78) | 1891/5444 | 60/113 | 2.45 (1.67 ~ 3.59) | <.001 |  |
| Yes | 1238 (18.22) | 492/1010 | 120/228 | 1.74 (1.25 ~ 2.43) | 0.001 |  |
| Hypertension, n(%) |  |  |  |  |  | 0.369 |
| No | 2126 (31.29) | 443/2038 | 31/88 | 2.14 (1.18 ~ 3.88) | 0.012 |  |
| Yes | 4669 (68.71) | 1940/4416 | 149/253 | 2.03 (1.50 ~ 2.75) | <.001 |  |
| **Diabetes mellitus**, n(%) |  |  |  |  |  | 0.355 |
| No | 4252 (62.58) | 1366/4082 | 76/170 | 1.73 (1.16 ~ 2.59) | 0.008 |  |
| Yes | 2543 (37.42) | 1017/2372 | 104/171 | 2.53 (1.80 ~ 3.56) | <.001 |  |
| Hyperlipidemia, n(%) |  |  |  |  |  | 0.203 |
| No | 1277 (18.79) | 368/1199 | 41/78 | 1.86 (1.11 ~ 3.13) | 0.019 |  |
| Yes | 5518 (81.21) | 2015/5255 | 139/263 | 1.90 (1.39 ~ 2.60) | <.001 |  |
| Sex, n(%) |  |  |  |  |  | 0.286 |
| Male | 2379 (35.01) | 227/2252 | 24/127 | 2.94 (1.51 ~ 5.71) | 0.002 |  |
| Female | 4416 (64.99) | 2156/4202 | 156/214 | 2.19 (1.63 ~ 2.93) | <.001 |  |
| Adjusted: ALT, AST, Ca, HCO3, GGT, Glu, TP, TG, UA, Na, K, Cl, MonP, EoP, BaP, Mon, Eo, Ba, RBC, Hem, MCH, MCHC, MPV, HDL, BMI. RAR, red cell distribution width-to-albumin ratio; PIR, poverty income ratio; HR: Hazard Ratio, CI: Confidence Interval | | | | | | |
