# Supplementary material for: Red cell distribution width-to-albumin ratio and chronic kidney disease mortality in adults: A population-based NHANES 1999 to 2020 study
Source: Medicine (Baltimore). 2026 Jun 12;105(24):e44559. doi: 10.1097/MD.0000000000044559 (PMC13268450; doi:10.1097/MD.0000000000044559)
Supplement: Supplementary file 8 [file medi-105-e44559-s008.docx]

Table S7. Regression analysis of exposure and outcome (NLR model)

| Variables | β | SE | Z | P | HR (95.CI) |
| --- | --- | --- | --- | --- | --- |
| RAR | 0.62 | 0.07 | 9.31 | <.001 | 1.87 (1.64 - 2.13) |

### RAR, red cell distribution width-to-albumin ratio; HR, hazard ratio; CI, confidence interval.
