# Supplementary material for: Red cell distribution width-to-albumin ratio and chronic kidney disease mortality in adults: A population-based NHANES 1999 to 2020 study
Source: Medicine (Baltimore). 2026 Jun 12;105(24):e44559. doi: 10.1097/MD.0000000000044559 (PMC13268450; doi:10.1097/MD.0000000000044559)
Supplement: Supplementary file 9 [file medi-105-e44559-s009.docx]

Table S8. Regression analysis of exposure, mediator and outcome (NLR model)

| Variables | β | SE | Z | P | HR (95%CI) |
| --- | --- | --- | --- | --- | --- |
| RAR | 0.56 | 0.07 | 8.06 | <.001 | 1.76 (1.53 - 2.02) |
| NLR | 0.13 | 0.02 | 8.26 | <.001 | 1.13 (1.10 - 1.17) |

### RAR, red cell distribution width-to-albumin ratio; NLR, neutrophil-to-lymphocyte ratio; HR, hazard ratio; CI, confidence interval.
