# Supplementary material for: Red cell distribution width-to-albumin ratio and chronic kidney disease mortality in adults: A population-based NHANES 1999 to 2020 study
Source: Medicine (Baltimore). 2026 Jun 12;105(24):e44559. doi: 10.1097/MD.0000000000044559 (PMC13268450; doi:10.1097/MD.0000000000044559)
Supplement: Supplementary file 10 [file medi-105-e44559-s010.docx]

Table S9. Mediation analysis: Effect estimates (NLR model)

| Effect | Estimate | Lower | Upper | β (95%CI) | P | Mediation |
| --- | --- | --- | --- | --- | --- | --- |
| Indirect | -3.18 | -4.32 | -1.77 | -3.18 (-4.32 ~ -1.77) | <.001 | 7.89 |
| Direct | -37.66 | -43.59 | -30.38 | -37.66 (-43.59 ~ -30.38) | <.001 | 92.11 |
| Total | -40.84 | -47.52 | -33.60 | -40.84 (-47.52 ~ -33.60) | <.001 | 100.00 |

### CI, confidence interval.
