# Supplementary material for: Red cell distribution width-to-albumin ratio and chronic kidney disease mortality in adults: A population-based NHANES 1999 to 2020 study
Source: Medicine (Baltimore). 2026 Jun 12;105(24):e44559. doi: 10.1097/MD.0000000000044559 (PMC13268450; doi:10.1097/MD.0000000000044559)
Supplement: Supplementary file 11 [file medi-105-e44559-s011.docx]

Table S10. Mediation analysis: Path analysis (NLR model)

| Path | Relationship | β | SE | Lower | Upper | P | β (95%CI) |
| --- | --- | --- | --- | --- | --- | --- | --- |
| RAR -> NLR | Exposure -> Mediator | 0.49 | 0.07 | 0.36 | 0.62 | <.001 | 0.49 (0.36 ~0.62) |
| RAR ->mortstat | Exposure -> Outcome | 0.56 | 0.07 | 0.43 | 0.70 | <.001 | 0.56 (0.43 ~ 0.70) |
| NLR -> mortstat | Mediator -> Outcome | 0.13 | 0.02 | 0.10 | 0.16 | <.001 | 0.13 (0.10 ~ 0.16) |

### RAR, red cell distribution width-to-albumin ratio; NLR, neutrophil-to-lymphocyte ratio; HR, hazard ratio; CI, confidence interval.
