# Supplementary material for: Red cell distribution width-to-albumin ratio and chronic kidney disease mortality in adults: A population-based NHANES 1999 to 2020 study
Source: Medicine (Baltimore). 2026 Jun 12;105(24):e44559. doi: 10.1097/MD.0000000000044559 (PMC13268450; doi:10.1097/MD.0000000000044559)
Supplement: Supplementary file 12 [file medi-105-e44559-s012.docx]

Table S11. Regression analysis of exposure and mediator (SII model)

| Variables | β | SE | t | P | β(95%CI) |
| --- | --- | --- | --- | --- | --- |
| Intercept | 3,966.85 | 1,077.07 | 3.68 | <.001 | 3966.85 (1855.83 ~ 6077.88) |
| RAR | 140.08 | 21.93 | 6.39 | <.001 | 140.08 (97.11 ~ 183.06) |

### RAR, red cell distribution width-to-albumin ratio; CI, confidence interval.
