# Supplementary material for: Red cell distribution width-to-albumin ratio and chronic kidney disease mortality in adults: A population-based NHANES 1999 to 2020 study
Source: Medicine (Baltimore). 2026 Jun 12;105(24):e44559. doi: 10.1097/MD.0000000000044559 (PMC13268450; doi:10.1097/MD.0000000000044559)
Supplement: Supplementary file 14 [file medi-105-e44559-s014.docx]

Table S13. Regression analysis of exposure, mediator and outcome (SII model)

| Variables | β | SE | Z | P | HR (95.CI) |
| --- | --- | --- | --- | --- | --- |
| RAR | 0.60 | 0.07 | 8.64 | <.001 | 1.82 (1.59 - 2.08) |
| SII | 0.01 | 0.00 | 4.01 | <.001 | 1.01 (1.01 - 1.01) |

### RAR, red cell distribution width-to-albumin ratio; SII, systemic immune-inflammatory index; HR, hazard ratio; CI, confidence interval.
