# Supplementary material for: Red cell distribution width-to-albumin ratio and chronic kidney disease mortality in adults: A population-based NHANES 1999 to 2020 study
Source: Medicine (Baltimore). 2026 Jun 12;105(24):e44559. doi: 10.1097/MD.0000000000044559 (PMC13268450; doi:10.1097/MD.0000000000044559)
Supplement: Supplementary file 15 [file medi-105-e44559-s015.docx]

Table S14. Mediation analysis: Effect estimates (SII model)

| Effect | Estimate | Lower | Upper | β (95%CI) | P | Mediation |
| --- | --- | --- | --- | --- | --- | --- |
| Indirect | -2.1 | -3.40 | -0.78 | -2.10 (-3.40 ~ -0.78) | <.001 | 5.27 |
| Direct | -39.0 | -44.91 | -31.42 | -39.00 (-44.91 ~ -31.42) | <.001 | 94.73 |
| Total | -41.1 | -47.75 | -33.82 | -41.10 (-47.75 ~ -33.82) | <.001 | 100.00 |

### CI, confidence interval.
