# Supplementary material for: Red cell distribution width-to-albumin ratio and chronic kidney disease mortality in adults: A population-based NHANES 1999 to 2020 study
Source: Medicine (Baltimore). 2026 Jun 12;105(24):e44559. doi: 10.1097/MD.0000000000044559 (PMC13268450; doi:10.1097/MD.0000000000044559)
Supplement: Supplementary file 16 [file medi-105-e44559-s016.docx]

Table S15. Mediation analysis: Path analysis (SII model)

| Path | Relationship | β | SE | Lower | Upper | P | β (95%CI) |
| --- | --- | --- | --- | --- | --- | --- | --- |
| RAR -> SII | Exposure -> Mediator | 140.08 | 21.93 | 97.11 | 183.06 | <.001 | 140.08 (97.11 ~183.06) |
| RAR ->mortstat | Exposure -> Outcome | 0.60 | 0.07 | 0.46 | 0.73 | <.001 | 0.60 (0.46 ~ 0.73) |
| SII -> mortstat | Mediator -> Outcome | 0.01 | 0.00 | 0.00 | 0.00 | <.001 | 0.01 (0.00 ~ 0.00) |

RAR, red cell distribution width-to-albumin ratio; SII, systemic immune-inflammatory index; HR, hazard ratio; CI, confidence interval.
